# Electron‐Lattice Synergistic Coordination for Boosting the Electro‐Optic Response of the Crystal

**DOI:** 10.1002/advs.202411143

**Published:** 2024-12-05

**Authors:** Jinfeng Han, Yuzhou Wang, Fei Liang, Kui Wu, Dazhi Lu, Deliang Cui, Haohai Yu, Huaijin Zhang

**Affiliations:** ^1^ State Key Laboratory of Crystal Materials and Institute of Crystal Materials Shandong University Jinan 250100 P. R. China; ^2^ The 46th Research Institute China Electronics Technology Group Corporation Tianjin 300220 P. R. China

**Keywords:** broadband electro‐optic modulation, electro‐optic response, electron‐lattice synergistic coordination strategy, langasite family crystal

## Abstract

The electro‐optic (E‐O) response is fundamental where the refractive index of materials can be modulated by the external electric field. Up to now, E‐O modulation has become a critical technology in photonics, quantum optics, modern communication, etc. However, the design of E‐O crystals with broadband availabilities and large E‐O effect is still challenging since the E‐O response is contributed by both electrons and phonons. Here, an electron‐lattice synergistic coordination strategy is proposed for boosting the E‐O response, and with the langasite as the subject, a novel crystal La_3_Zr_0.5_Ga_5_Si_0.5_O_14_ (LZGS) is designed. By the rational introduction of the Zr^4+^ ion in the octahedral site, it is found that the E‐O response can be obviously improved attributed to the active 4*d* orbitals of the Zr^4+^ ion and the optical phonons of the flexible (ZrO_6_) unit. This crystal also has advantages in transparency, laser damage threshold and E‐O coefficient compared with the well‐known E‐O crystals. Moreover, broadband E‐O modulation is also demonstrated in the lasers at wavelengths from 639 to 1991 nm. These findings not only demonstrate an excellent E‐O crystal for the development of modern devices in classical and quantum fields but also provide a design strategy for boosting the E‐O response.

## Graphical abstract

This work proposes an electron‐lattice synergistic coordination strategy for boosting the E‐O response, and with the langasite as the subject, a novel crystal La_3_Zr_0.5_Ga_5_Si_0.5_O_14_ (LZGS) is designed with good electro‐optic properties. Moreover, broadband E‐O modulation of LZGS is also demonstrated in the lasers at wavelengths from 639 to 1991 nm.

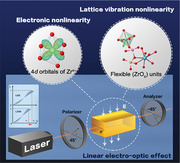

## 1 Introduction

The interaction between light and matter is a fundamental issue in optics, material science, and even physics and chemistry.^[^ ^1^, ^2^, ^3^ ^]^ Under the external field, the optical response of materials would be changed, and therefore, the light performance could be modulated through the interaction process. Associated with the modulation of an external field, the light feature including the phase, frequency, polarization and even transmission directions could be manipulated, which lays the foundation of classical and quantum optics.^[^ ^4^, ^5^, ^6^, ^7^ ^]^ The electric field is extensively applied to the external field, and under which the refractive index of materials would be perturbed, corresponding to the manipulation of light states by the eletro‐optic (E‐O) effect.^[^ ^8^, ^9^ ^]^ Up to now, E‐O modulation has become a critical technology in the optical network, integrated optics, high‐intense lasers, etc., and the E‐O modulators have been successfully applied as modern devices, including isolators, frequency modulators, beam splitters, and even Q‐switchers.^[^ ^10^, ^11^, ^12^, ^13^, ^14^, ^15^ ^]^ However, the E‐O materials with excellent properties still need to be investigated, and the rational design of E‐O crystals is still challenging since the E‐O response is contributed by the charge movements, optical and acoustic branches of lattice vibrations under the external electrical fields.^[^ ^16^, ^17^, ^18^ ^]^ The enhancement of a certain item mentioned above would always generate a destructive effect on others due to the correlation.^[^ ^19^, ^20^, ^21^, ^22^ ^]^

Especially with the development of modern communications with high capacities and laser medicine with high precisions, broadening wavelength bands toward the mid‐infrared requires E‐O modulators with broadband availabilities, high damage thresholds and large E‐O effects.^[^ ^23^, ^24^, ^25^, ^26^, ^27^ ^]^ Besides, the favorable E‐O modulators also require materials with low piezoelectric effects since the piezoelectric would generate additional microcosmic mechanical movement and affect the E‐O performance.^[^ ^28^, ^29^ ^]^ Although some quantitative calculation methods for E‐O materials have been proposed in the past tens of years,^[^ ^30^, ^31^, ^32^ ^]^ the multiple correlated mechanisms shown above constrain the development of E‐O materials. The discovery of new E‐O materials with highly comprehensive properties is rarely reported, which also restricts the development of further related application areas. Here, associated with the E‐O theory, we propose an electron‐lattice synergistic coordination strategy for boosting the electro‐optic response, where the electron and phonon contributions in a critical site are enhanced simultaneously but shield the destruction in the neighbors. With the langasites as the subject, we design a novel crystal La_3_Zr_0.5_Ga_5_Si_0.5_O_14_ (LZGS) with the advantages of transparency, laser damage threshold, and E‐O coefficient. It could be believed that this work provides novel E‐O material for modern applications and some inspiration for the design of functional materials as well.

## 2 Results and Discussion

### 2.1 Electron‐Lattice Synergistic Coordination Strategy

As mentioned above, the E‐O response is contributed by charge movements and lattice vibrations according to the E‐O theory.^[^ ^16^, ^17^, ^18^ ^]^ The cooperative increase of the electron and lattice vibration contributions is challenging when designing E‐O crystals, since a destructive effect on others would appear when enhancing a certain item.^[^ ^19^, ^20^, ^21^, ^22^ ^]^ In order to solve this problem, here we propose to rationally introduce a suitable cation at a given symmetric site in a crystal, thereby regulating the electronic orbitals and projected phonon‐state density simultaneously but shielding the destruction in the neighbors. Consequently, both the electron and lattice contributions give the same sign for the Pockels coefficient, named electron‐lattice synergistic coordination strategy. For this purpose, the as‐designed crystal structure should 1) be non‐centrosymmetric for achieving the second‐order nonlinear property, 2) be composed of multiple sites or groups with a high tolerance to provide the capacity of enhancing one certain property (such as the contribution of the lattice vibration) without generating destructive effects on other properties related to the structure (for example the nonlinearity, transparent range and laser damage threshold, etc.). In the meantime, the substituted cation should be sensitive to the electric field response, which is conducive to the charge movements and lattice vibration to contribute to the E‐O response.

**Figure 1:**
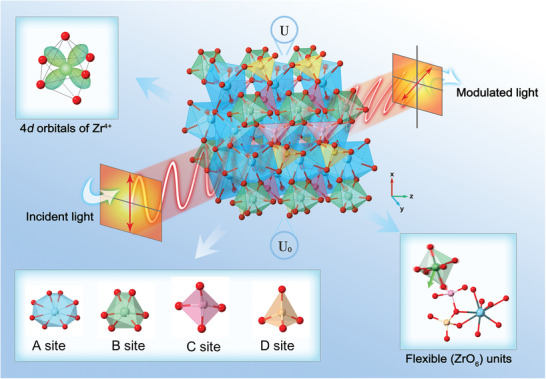
Schematic diagram for E‐O crystal design with an electron‐lattice synergistic coordination strategy. The linear E‐O coefficient of langasite compounds is mainly determined by the electronic nonlinearity and lattice nonlinearity via introducing Zr^4+^ ion at (BO_6_) polyhedron.

As the E‐O crystals, the langasite family belongs to the *P321* space group and can be described using a general chemical formula A_3_BC_3_D_2_O_14_. Langasite crystal has four ionic occupation sites: dodecahedral A site, octahedral B site, and tetrahedral C and D site, respectively.^[^ ^33^ ^]^ In previous studies, we have deciphered that the second‐order nonlinearity of langasite is mainly contributed by the induced susceptibility of electronic orbitals in (AO_8_) and (BO_6_) units.^[^ ^34^ ^]^ Nevertheless, this case becomes more complicated when improving the linear E‐O effect based on this crystal structure, since lattice vibrations also make a crucial contribution to the E‐O coefficient besides the induced polarizability from electron response,^[^ ^35^ ^]^ as presented in **Figure** **1**. Herein, according to the design strategy and precautions proposed above, the B site is selected to be adjusted by cation substitution for optimizing the linear E‐O coefficient of the crystal, based on the following considerations. First, the (AO_8_) dodecahedron is maintained since it contributes the most among the four groups both in second harmonic generation (SHG) and E‐O effects. For example, the (LaO_8_) anionic groups contribute more than 80% to the overall SHG coefficient in La_3_Ga_5_SiO_14_ (LGS),^[^ ^34^ ^]^ and the E‐O coefficient *γ_11_* of lanthanum‐free Ca_3_TaGa_3_Si_2_O_14_ (CTGS) is 14 times smaller than that of LGS.^[^ ^36^ ^]^ Second, the contributions of both the (CO_4_) and (DO_4_) tetrahedra are negligible and the second‐order nonlinearity is cancelled out due to the site symmetry.^[^ ^34^ ^]^ Third, the B site is more “flexible” in response to the external electric field,^[^ ^34^, ^37^, ^38^, ^39^, ^40^, ^41^ ^]^ and is more tolerant due to the provided suitable neighboring conditions for shielding the destruction during the cation substitution, since the dodecahedron and tetrahedra exhibit strong and negligible responses to the external field, respectively.

Thereupon, the Zr^4+^ cation is incorporated into the B site, forming a novel crystal La_3_Zr_0.5_Ga_5_Si_0.5_O_14_ (LZGS), under the following considerations: i) The 4*d* orbitals of Zr^4+^ are sensitive to electric field response,^[^ ^42^ ^]^ indicating that the (ZrO_6_) units are advantageous for improving dielectric performance and then induce high nonlinear polarizability. ii) The Zr^4+^ cation holds a sizeable atomic mass, which helps reduce the vibrational frequency, thereby extending the transmittance of crystal in the infrared range. iii) The ionic radius of Zr^4+^ (*r* ≈ 0.72 Å) is comparable with that of Ga^3+^ (*r* ≈ 0.62 Å), which is suitable for occupying the B site in langasite crystal and reduces the structure disorder in the small‐radius C and D sites.

### 2.2 Synthesis and Characterization of LZGS Polycrystalline

Guided by the above design strategy, we synthesized the LZGS polycrystalline by the high‐temperature solid‐state reaction. The single‐crystal X‐ray diffraction showed that LZGS crystallizes in the *P321* space group. The XRD pattern was consistent with the standard PDF card without any impurities (Figure S1, Supporting Information).

**Figure 2:**
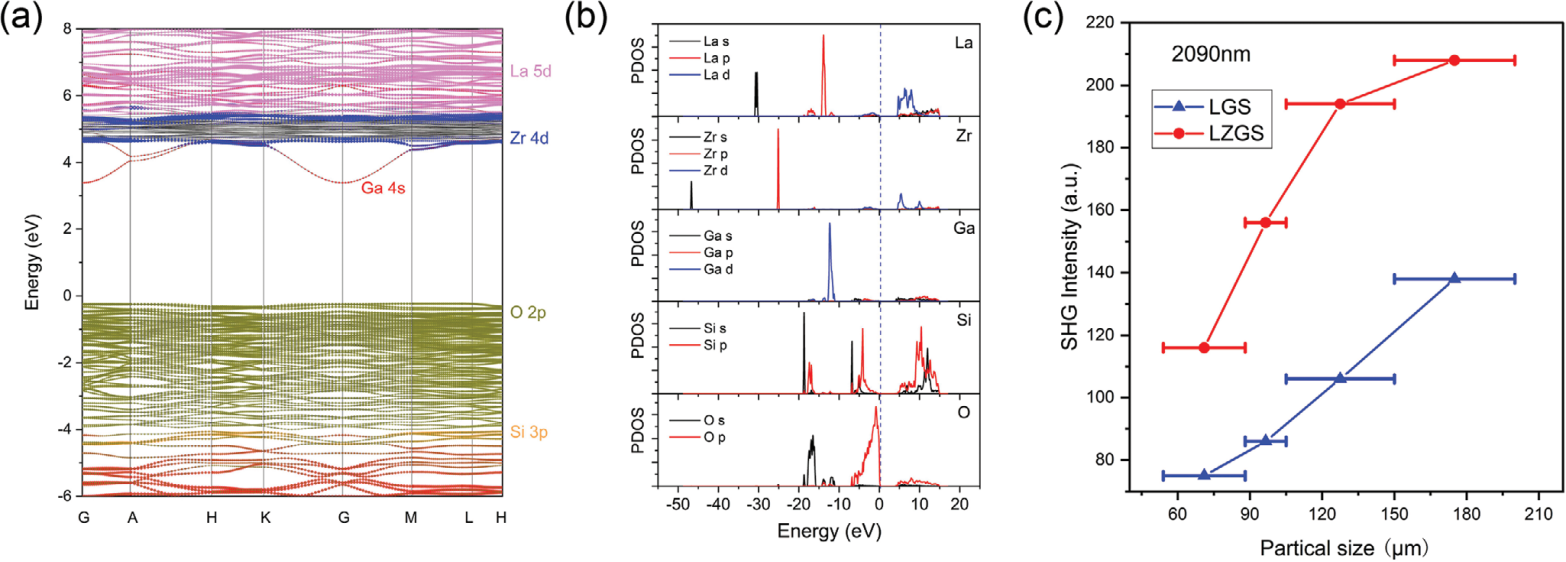
a) Calculated electronic band structure of LZGS. b) The partial density of state density of LZGS. c) SHG responses of LZGS at 2090 nm. LGS crystal was plotted as a reference.

Based on the refined crystal structure of LZGS, we calculated its electronic band structure using first‐principle calculations. As shown in **Figure** **2**a, it was an indirect‐gap crystal with a simulated forbidden gap value of 3.63 eV. The projected electronic orbitals showed that the O 2*p* electrons mainly occupy the upper valence band, whereas the bottom conduction band was contributed from Ga 4*s*, Zr 4*d*, and La 5*d* orbitals. Figure 2b depicted the partial density of state density of the constituent atoms of LZGS. We could see that the 5*d* orbitals of La and the 4*d* orbitals of Zr contributed the most to the conduction band minimum region. As we know, the optical response of a crystal is predominantly determined by the optical transition between the electronic states near the forbidden gap. As a result, the introduced Zr^4+^ cation would make a constructive contribution to the induced nonlinear polarizability and corresponding E‐O coefficients, which is consistent with our design strategy.

In order to clarify the positive role of (ZrO_6_) for the E‐O effect of LZGS, we calculated the flexibility index^[^ ^43^ ^]^ of (ZrO_6_) in the LZGS crystal and (GaO_6_) in the LGS crystal (detailed calculation method was listed in Supporting Information). This factor can be applied to prejudge the contribution of (MO_6_) octahedra to the induced dipole polarizability, as well as the corresponding linear E‐O coefficient. The flexibility index of octahedra [(Zr/Ga)O_6_] (*F* = 0.1313) in LZGS is more significant than that of (GaO_6_) (*F* = 0.1114) in the LGS (Table S1, Supporting Information), which indicated that (ZrO_6_) is conducive to improve the structural flexibility and further regulate the E‐O coefficient.

Then, we made preliminary quantitative calculations for the linear E‐O coefficient of LZGS based on the powder method reported by Ye et al.^[^ ^17^ ^]^ in experiments. The contributions of charge movements to the E‐O coefficient could be characterized by the SHG measurement. As shown in Figure 2c, the powder SHG intensity of LZGS polycrystalline was measured by the Kurtz–Perry method, with a strong phase‐matchable SHG efficiency of 1.5 × LGS. On the basis of the first‐principle calculations and SHG measurements, we have preliminarily verified the feasibility of the crystal design in terms of electronic nonlinearity.

**Figure 3:**
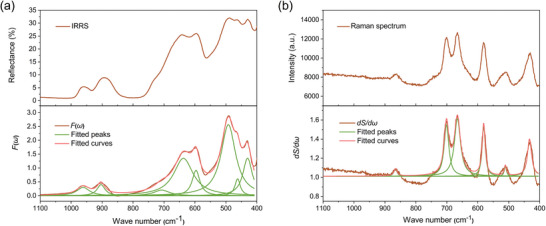
a) The IRRS and *F(ω)*, b) Raman spectrum and *dS/dω* of LZGS powder.

The contributions of lattice vibrations to the E‐O coefficient were evaluated by the infrared reflectance spectrum (IRRS) and Raman spectrum,^[^ ^17^ ^]^ which can be represented by the dipole transition matrix element *M(r)* and the transition‐susceptibility matrix element *P(r)*. The *F(ω)* and *dS/dω* had the specific calculation relationship with *M(r)* and *P(r)*, respectively (detailed calculation methods were listed in Supporting Information). LZGS powders were utilized in all measurements, and LGS powders were also measured as a reference. The IRRS and Raman spectrum, fitted peaks, and fitted curve of LZGS powders were shown in **Figure** **3**, and the *M(r)* and *P(r)* values corresponding to each fitting peak were listed in Tables S2 and S3 (Supporting Information), respectively. The calculated values at the same center frequency were listed in Table S4 (Supporting Information). For LZGS crystal, the calculated contribution of lattice vibrations to the E‐O coefficient was derived as 3.99 pm·V^−1^ through the proposed powder E‐O measurement method. Under the same conditions, the contributions of lattice vibrations in LGS for the E‐O coefficient was only 1.33 pm·V^−1^ (more detailed data were shown in Figure S2 and Tables S5–S7, Supporting Information). Therefore, in terms of lattice vibrations, we also verified the constructive contribution of the introduced Zr^4+^ cation to the increased linear E‐O effect.

### 2.3 Growth and E‐O Modulation Demonstration of LZGS Crystal

Based on the above calculations and analysis, it was preliminarily confirmed that introducing Zr^4+^ at the B site is favorable for increasing the E‐O coefficient. Then, the growth of large‐size and high‐quality LZGS crystal is performed under the optimized growth conditions. The crystal was grown using the Czochralski method. After a series of components (Figure S3, Supporting Information), atmosphere (Figure S4, Supporting Information) and temperature field optimization (Figure S5, Supporting Information), a large LZGS crystal with a diameter of 25 and 22 mm in length (Figure S6, Supporting Information) was grown successfully. A more detailed optimization process was listed in Supporting Information.

The crystalline quality of an electro‐optic crystal is vital for practical application. Thereby, the rocking curve and conoscopic interference patterns have been both characterized for evaluating the crystal crystallinity and optical uniformity of the grown LZGS crystal, respectively. Figure S7 (Supporting Information) presented the rocking curves of the (0001) and $\left(11\bar{2}0\right)$ wafers cut from the body parts of the LZGS crystal. Their full‐width at half‐maximum (FWHM) values were measured to be 23.9″ and 27.4″, indicating the high crystal crystallinity. The conoscopic interference experiments were carried out using three samples cut along *c*‐axis from the top, middle and bottom parts of the as‐grown crystal. As presented in Figure S8 (Supporting Information), the light and dark interferences were distributed regularly and smoothly, and interference patterns were highly symmetrical, indicating the fine optical uniformity of the as‐grown crystal.

**Figure 4:**
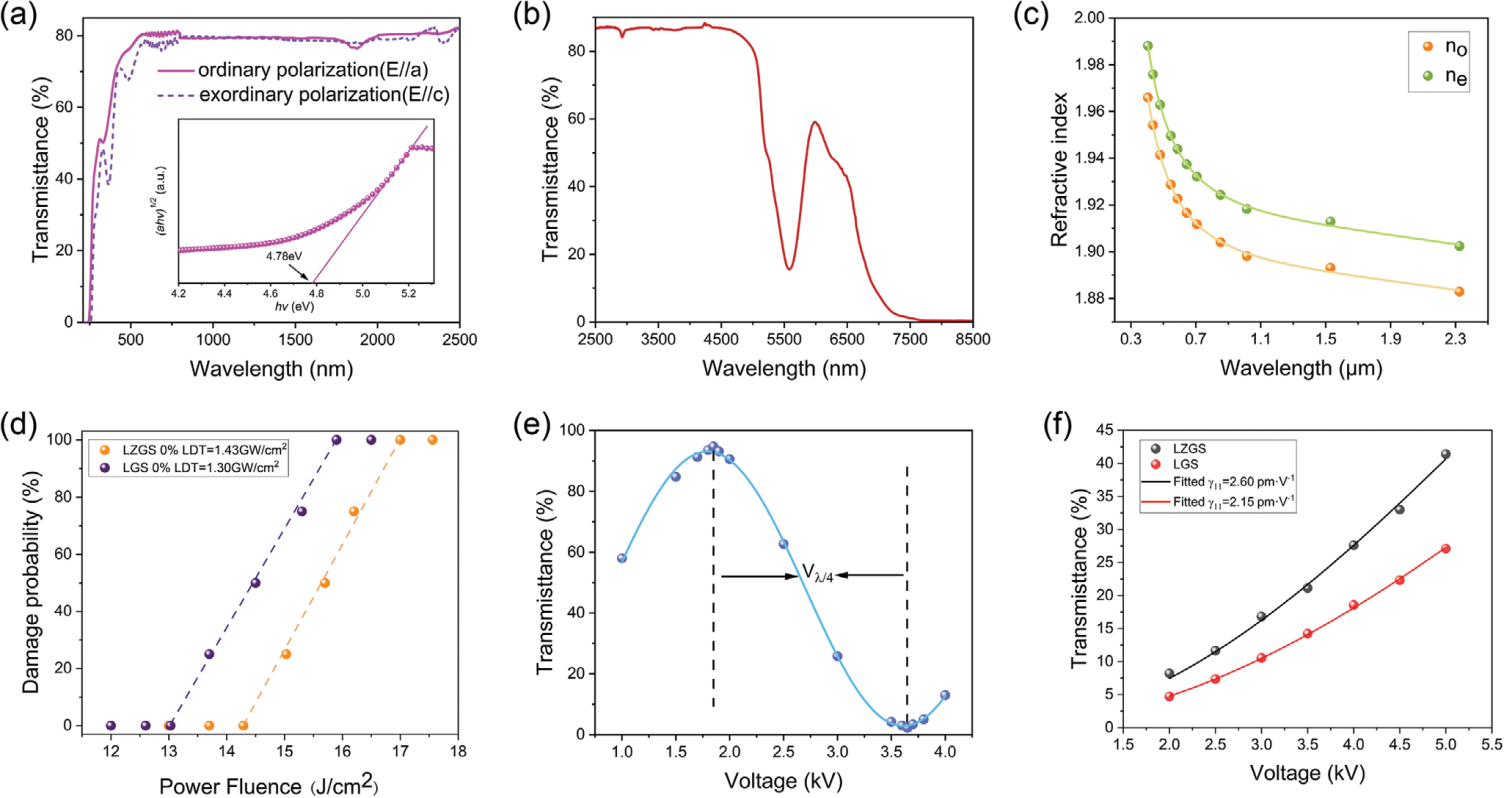
a) Polarized UV−vis transmission spectra of LZGS and the inset of (a) are the (*αhv*)^1/2^ versus *hv* curves for determining the UV cut‐off. b) Unpolarized IR transmission spectra of LZGS. c) Measured principal refractive indices *n_o_* and *n_e_* plotted as a function of wavelength (dots), and fit of these experimental data (solid lines). d) The laser damage thresholds of the LZGS and LGS crystal. e) The relational graph of the light intensity transmittance with the voltage at 633 nm based on the *γ_11_* of LZGS crystal. f) The relational graph of the light intensity transmittance with the voltage at 1991 nm lasers based on the *γ_11_* of LZGS crystal.

The fundamental optical properties of LZGS were characterized in detail. The transmission spectra of LZGS crystal were measured over the UV‐vis‐IR region **(Figure** **4**a,b). LZGS had a wide transmission range from 260 to 7600 nm, completely covering the 3–5 µm atmospheric window. A multi‐phonon absorption peak at 5.5 µm was observed, which is assigned to the high‐frequency Si─O bond vibrations.^[^ ^44^ ^]^ Its optical bandgap was calculated using Tauc's equation^[^ ^45^ ^]^ *αhv* = A·(*hv* − E_g_)^2^ where *α* is the absorption coefficient and A is an energy‐independent constant. The (*αhv*)^1/2^ versus *hv* was plotted in the inset of Figure 4a, then the indirect bandgap was found by extrapolating the linear portion to horizontal intercept (*αhv*)^1/2^ = 0. The optical bandgap of LZGS was calculated to be 4.78 eV, and the corresponding UV cut‐off wavelength was 260 nm. Those values are comparable to those of LGS,^[^ ^44^ ^]^ indicating that introducing Zr^4+^ does not shorten the transparent range in the UV to near‐IR range.

The refractive index is indispensable for calculating nonlinear phase‐matching and E‐O rotation.^[^ ^46^ ^]^ LZGS crystal is a positive uniaxial crystal with two different refractive indices, ordinary and extraordinary refractive index *n_o_* and *n_e_* (*n_o_* < *n_e_*). The measured refractive indices of LZGS as a function of the wavelength were fitted using the least‐squares method according to the Sellmeier equation,^[^ ^39^ ^]^ *n* ^2^ = A + B / (*λ*_2_ – C) – D*λ*_2_, where *λ* is the wavelength in µm unit and A, B, C, D are the parameters. As shown in Figure 4c, the measured refractive indices agreed well with the fitted Sellmeier equation. The fitted Sellmeier equation was expressed as:

(1) $$n_{e}^{2}\left(\lambda \right)=3.65079+\frac{0.04075}{\lambda^{2}-0.02963}-0.00690\lambda^{2}$$

(2) $$n_{o}^{2}\left(\lambda \right)=3.57539+\frac{0.03893}{\lambda^{2}-0.03033}-0.00655\lambda^{2}$$

We compared the refractive indices of LZGS and LGS at the same wavelength, as shown in Table S8 (Supporting Information). It can be seen that the *n_o_* and *n_e_* of LZGS crystal at the same wavelength were larger than the *n_o_* and *n_e_* of LGS, and the birefringence (∆*n* = 0.02 at 1014 nm) of LZGS was also slightly larger than the birefringence (∆*n* = 0.01) of LGS. Because the driving voltage is inversely proportional to the refractive index *n*, the increased refractive index *n* is conducive to reducing the drive voltage.

In addition, the laser damage thresholds of LZGS and LGS crystals were measured. As shown in Figure 4d, when the power fluence was set as 14.3 J·cm^−2^ at 1064 nm, with 1 mm of the beam diameter, there was no surface damage appeared on the LZGS crystal, which corresponded to a 0% laser damage of 1.43 GW·cm^−2^. Under the same conditions, the 0% laser damage of LGS crystal was 1.30 GW·cm^−2^.

The nonlinear coefficients of LZGS crystal were measured by the Marker–Fringe method,^[^ ^47^ ^]^ and the Type‐I SHG conversion was selected. LZGS has only one independent nonlinear coefficient *d*_11_, which could be compared with *d*_36_ of KDP crystals with (*x, y*) plane to determine the nonlinear coefficient of LZGS. The polarization directions of the fundamental beam, *E_ω_*, and the detected second harmonic beam, *E_2ω_*, were worked out and diagrammed in Figure S9 (Supporting Information). As shown in Figure S10 (Supporting Information), the measured *d*_11_ Maker fringe pattern of LZGS crystal was in good agreement with the theoretical pattern, and the second‐order nonlinear coefficient *d*_11_ of LZGS crystal was calculated to be 3.16 pm V^−1^, which is significantly larger than that of LGS^[^ ^44^ ^]^ (*d*_11_ = 1.7 pm·V^−1^).

LZGS has two independent linear E‐O coefficients, *γ_11_* and *γ_41_*. Considering that the transverse E‐O effect can reduce the drive voltage and based on the E‐O coefficient matrix, we chose the Z‐cut crystal to measure the E‐O coefficient *γ_11_*. Therefore, the E‐O coefficient *γ_11_* of LZGS crystal was measured using *V_π/2_* method at 633 nm He‐Ne laser. The experimental configuration was shown in the in Figure S11 (Supporting Information). The relationship between the light intensity transmittance and the voltage was shown in Figure 4e. When the external voltage was applied to the LZGS crystal, the quarter‐wave voltage was 1.83 kV, and the calculated E‐O coefficient *γ_11_* of the LZGS crystal was 2.55 pm·V^−1^ (detailed measurements were listed in Supporting Information). In contrast, the E‐O coefficient of LGS crystal was 2.18 pm·V^−1^ under the same condition, suggesting that our proposed electron‐lattice synergistic coordination strategy effectively increases the E‐O coefficients of the langasite family.

In order to further measure the linear E‐O coefficient of LZGS crystal in the mid‐infrared band, we built a 1991 nm laser device, as shown in Figure S12 (Supporting Information), and we simultaneously prepared LGS crystal for comparison. Linearly polarized light passing through LZGS and LGS crystal was rotated 20° and 5° about the optical *c* axis, respectively. The relationship between the light intensity transmittance and the voltage was shown in Figure 4f. The E‐O coefficient *γ_11_* of LZGS crystal was 2.6 pm·V^−1^, and that of LGS crystal was 2.15 pm·V^−1^ (detailed measurement method was listed in Supporting Information). The experimental results were comparable to those obtained under a 633 nm light source. The rationality of the proposed new E‐O crystal design strategy was further verified through the E‐O coefficient characterization.

We also characterized the piezoelectric properties of the LZGS crystal. The measured results were shown in Table S9 (Supporting Information). The piezoelectric coefficients of LZGS were smaller than those of LGS crystal, and its piezoelectric coefficients *d*_11_ (6.1 pC·N^−1^) and *d*_14_ (‐5.1 pC·N^−1^) were nearly ten times smaller than that of LiNbO_3_^[^ ^48^ ^]^ crystal (*d*_15_ = 67.7 pC·N^−1^), which is conducive to restraining piezoelectric ringing effect in the E‐O system. In addition, the room‐temperature resistivity of LZGS crystal were measured, with *ρ*_11_ = 1.08×10^12^ Ω·cm and *ρ*_33_ = 1.24×10^12^ Ω·cm.

The results of LZGS have been summarized as shown in Table S10 (Supporting Information). LZGS has the widest transmission range of all practical E‐O crystals, which can ensure its use in the mid‐infrared band. In addition, it has excellent resistivity and piezoelectric properties to ensure the safety and stability of the E‐O modulation system. Compared to LiNbO_3_ crystal, LZGS crystal has a high laser damage threshold, which is capable of transmitting high‐power modulated lasers. In addition, compared with LGS crystal, the nonlinear optical coefficient and linear E‐O coefficient of LZGS crystals are increased by 80% and 20%, respectively, which verify the feasibility of the electron‐lattice synergistic coordination strategy.

**Figure 5:**
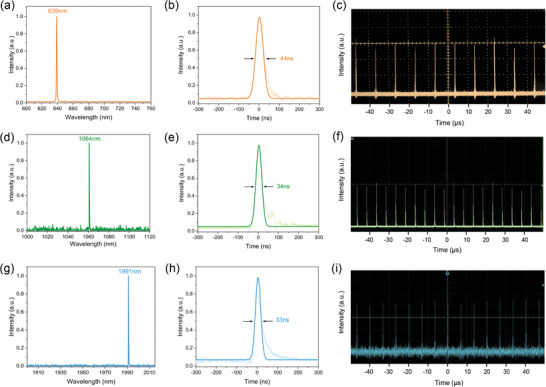
a) Spectrum of the LZGS E‐O Q‐switched pulsed laser at 639 nm. b) Pulse profile at 639 nm laser. c) Pulse train with a repetition rate of 100 kHz at 639 nm. d) Spectrum of the LZGS E‐O Q‐switched pulsed laser at 1064 nm. e) Pulse profile at 1064 nm laser. f) Pulse train with a repetition rate of 150 kHz at 1064 nm. g) Spectrum of the LZGS E‐O Q‐switched pulsed laser at 1991 nm. h) Pulse profile at 1991 nm laser. i) Pulse train with a repetition rate of 150 kHz at 1991 nm.

We further fabricated several E‐O modulators using high‐quality LZGS crystals, and demonstrated their broadband light modulation and pulsed laser generation capabilities. The specific schematic and experimental procedures were shown in the Supporting Information, and the experimental results were shown in **Figure** **5**. Figure 5a–c presented the generated pulsed laser with the LZGS E‐O Q‐switcher in the visible wavelength range, which had a central wavelength of 639 nm, a pulse width of 44 ns and a repetition rate of 100 kHz. This was the first report of pulsed laser output in the visible range using langasite E‐O Q‐switchers. Figure 5d–f presented the pulsed laser located at 1064 nm, with a pulse width of 34 ns and a repetition rate of 150 kHz. The quarter‐wave voltage of the LZGS E‐O switcher (with aspect ratio 5:1) at 1064 nm was 2950 V, which reduced by 15% (3400 V) when utilizing the LGS E‐O Q‐switcher with the same dimensions.^[^ ^49^ ^]^ Moreover, the highest repetition rate of the achieved pulsed laser was 150 kHz, which was much higher than that of LiNbO_3_ (7 kHz)^[^ ^50^ ^]^ and KDP (10 kHz).^[^ ^51^ ^]^ Then, in order to further verified the availability of LZGS crystal in the mid‐infrared band, the E‐O Q‐switching experiment was carried out for achieving the pulsed laser with a central wavelength of 1991 nm (Figure 5g), a pulse width of 33 ns (Figure 5h) and a repetition rate of 150 kHz (Figure 5i). Based on the high‐repetition‐rate pulsed lasers at wavelengths of 639, 1064, and 1991 nm without piezoelectric ringing effect, we believe that the LZGS E‐O Q‐switcher is capable to realize the mid‐infrared pulsed lasers under high drive voltages, revealing its potential application in the broadband and especially high repetition rate E‐O modulation, such as modern communication and laser medicine.

## 3 Conclusion

In conclusion, we proposed an electron‐lattice synergistic coordination strategy to boost the E‐O response of crystals with the cooperative increase of electron and phonon contributions. Under the guidance of the strategy, a novel LZGS crystal was designed based on the multifunctional langasite family crystals. Through the first‐principle calculations and vibrational spectrum measurements, the improvement of E‐O coefficient in the LZGS crystal is attributed to the active 4*d* orbitals of Zr^4+^ ion and optical phonons of flexible (ZrO_6_) unit. After comprehensive characterizations, the LZGS crystal has the advantages of transparency (0.26–7.6 µm), laser damage threshold (1.43 GW·cm^−2^), piezoelectric effect (*d*_11_ = 6.1 pC·N^−1^) and E‐O coefficient (2.6 pm·V^−1^), more advantages than the conventionally used E‐O crystal. Moreover, the piezoelectric coefficients of LZGS crystal were nearly ten times smaller than those of LiNbO_3_ crystal. After successfully growing a high‐optical‐quality crystal, the LZGS Pockels cell was demonstrated and utilized for achieving pulsed lasers at the wavelengths of 639, 1064, and 1991 nm, confirming its broadband E‐O modulation. All the experimental and theoretical results prove LZGS crystal a suitable E‐O material applied in the broadband modulation for the modern communications and laser medicine, etc. In addition, the as‐proposed electron‐lattice synergistic coordination strategy also paves the way for the effective exploration of new E‐O materials with highly comprehensive performance.

## 4 Experimental Section

### First‐Principle Calculations

The first‐principles tool‐CASTEP package^[^ ^52^ ^]^ were employed to perform all density functional theory (DFT)^[^ ^53^ ^]^ calculations. The ion‐electron interactions for all constituent elements were simulated by using the optimized norm‐conserving pseudopotential^[^ ^54^ ^]^ (See Supporting Information for detailed theoretical calculations).

### Polycrystalline Synthesis

La_2_O_3_, SiO_2_, ZrO_2_, and Ga_2_O_3_ powders with high quality (>99.99%) were purchased from Alfa Aesar and were weighed in the stoichiometric proportion. In order to maintain the consistency of the components, an extra 1.5 wt.% Ga_2_O_3_ was added to reduce volatilization in the growth process. The mixture is fully mixed in the mixer for 25 h and then pressed into blocks in the press. Finally, blocks are put into the muffle furnace for sintering. The sintering temperature of the muffle furnace is set at 1340 °C, and the holding time is 20 h so that it is a fully solid‐phase reaction, and the polycrystalline material is obtained. The solid‐phase reaction of this process proceeds according to the following chemical reaction:

(3)

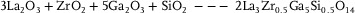




### Powder X‐Ray Diffraction Measurement

X‐ray diffraction analysis is the primary method for studying the phase and crystal structure of matter. To measure the XRD patterns of LZGS crystal powder, an automated Bruker D8 ADVANCE X‐ray diffractometer equipped with a diffracted monochromator set for Cu‐Kα (*λ* = 1.54056 Å) radiation was used. The measurements were performed in the angular range of 2*θ* = 10–70° with a scan step of 0.02°/0.4 s.

### Power SHG Measurement

SHG signals were measured using a Cr:Tm:Ho:YAG laser with a wavelength of 2.09 µm. First, the crystals were ground and sieved into four particle size ranges of 0.055–0.088, 0.088–0.105, 0.105–0.155, and 0.155–0.2 mm. LGS with the same particle size ranges were used as references. Second, they were placed on a microscope slide and held in place with transparent tape. Finally, the SHG signals were measured with the detector and recorded on an oscilloscope.

### Simulation of Temperature Field

The temperature distribution and crystal thermal stress inside the furnace were studied using STR CGSim software for insulation layers of different thicknesses.

### Single Crystal Growth

LZGS crystals were grown using the Czochralski method, which allowed larger crystals to be grown for later device design. The JPG Auto Diameter Control Program of the single‐crystal furnace automatically controlled the entire growth process, of which accuracy is ± 0.01 g. The crystals were grown using LGS seeds along the [001] direction in an iridium crucible with a diameter of 65 mm (500 g of the melt). Before seeding, the melt must be kept at a suitable temperature (20–30 °C higher than the melting point) for 8–12 h to establish uniform density, viscosity, surface tension and other physical and chemical properties of the melt. During the crystal growth process, each stage requires careful selection of different pulling velocities (0.6–1.0 mm·h^−1^) and rotation velocities (8–12 *rpm*) to ensure a slightly convex and stable solid–liquid interface shape.

### Transmission Spectrum Measurement

The transmission spectra were recorded using a 1 mm‐thick x‐cut slab with aperture dimensions of 5 × 7 mm^2^. The slab was uncoated and polished to optical quality. The transmission spectra in the visible and near IR range (0.2–2.5 µm) were then measured using a Cary 5000 DUV spectrophotometer emitting polarized light, and the spectra in the mid‐IR range (2.5–25 µm) were recorded on a Thermo‐Nicolet NEXUS 670 FTIR spectrophotometer emitting unpolarized light.

### Refractive Index Measurement

The high optical quality LZGS crystals were cut into prisms with a vertex angle of 18.54°. The (210) and (100) planes of the prisms were polished precisely without coating. The LZGS prism was placed in a high‐precision automatic spectrometer goniometer (HR Spectro Master UV–vis–IR from Trioptics). By adjusting the proper orientation of the linear polarization of the input beam, it has been possible to determine the values of the ordinary and extraordinary principal refractive indices of LZGS. Refractive indices (i.e.*, n_o_* and *n_e_*) of 10 sets of discrete wavelengths ranging from 0.4047 to 2.3250 µm were recorded with a precision of 10^−5^.

### Laser Damage Threshold Measurement

The LDTs were studied using a Q‐switched (Nd: YAG) laser at 1064 nm (LPS‐1064‐A) with a 10 ns pulse width and 1 Hz repetition rate. The (100) plane of the crystal was polished precisely without coating. The laser beam was focused onto a polished 1‐mm‐thick LZGS slab with aperture dimensions of 8 × 8 mm^2^ using a 200‐mm‐focal lens. The error in the laser energy was determined by combining the 3% uncertainties of the energy measurements. Laser conditioning was investigated as a function of frequency using the 1‐on‐1 approach.

### Maker‐Fringe Experiment

Using the Maker Fringe setup, type I second harmonic generation (SHG) was selected to determine *d*_11_ of LZGS relative to *d*_36_ of the KDP under identical wavelengths. For this purpose, the (*y, z*) plane of LZGS with dimensions of 5 × 5 × 1.0 mm^3^ was cut and polished to optical quality, while the (*x, y*) plane of KDP with dimensions of 5 × 5 × 1.0 mm^3^ was also prepared. An Nd:YAG Q‐switched laser with a pulse width of 10 ns and a repetition frequency of 10 Hz was employed as the fundamental light source. These samples were adhered to a turntable with a precision of 0.00125° (RAK 100, Zolix Inc). The second harmonic signal generated from the sample was detected using a side window photomultiplier tube (PMT), averaged using a fast‐gated integrator and boxcar averager (Stanford Research Systems), and automatically recorded by a computer.

### IRRS Spectroscopy

The IRRS in the 3000−400 cm^−1^ range was recorded on a Nicolet iS50 Fourier transform infrared spectrometer using an ATR device. The LZGS crystal was ground into uniform crystalline powders, and the sample's infrared reflectance was measured using the sphere integration method.

### Raman Spectroscopy

LZGS powders were placed on an objective slide, and an in situ confocal Raman Spectrometer (LabRAM HR Evolution) was used to record the Raman spectra under a laser excitation at 532 nm.

### Measurement of Piezoelectric Coefficient

LZGS crystals have two independent dielectric constants ε_11_ and ε_33_, and two independent piezoelectric strain constants *d*_11_ and *d*_14_. The dielectric constant was determined by the HP4294A impedance analyzer. The samples were 10 × 10× 1.5 mm^3^ square plates in the x and z directions. *d*_11_ was measured directly by the *d*_33_ quasi‐static tester, and *d*_14_ was measured by the resonant‐antiresonance method. The sample size of *d*_14_ was 2 × 4 × 1.6 mm^3^. The surfaces of the test wafer samples are uniformly coated with Platinum. LZGS and LGS were tested respectively under the same conditions.

### Measurement of E‐O coefficient

The LZGS crystal belonged to 32 point group in the trigonal system with two independent E‐O coefficients: *γ_11_* and *γ_41_*. LZGS is a uniaxial crystal without the electric field and the refractive‐index ellipsoid can be expressed as follows:

(4) $$\frac{1}{n_{o}^{2}}\times \left(x^{2}+y^{2}\right)+\frac{1}{n_{e}^{2}}\times z^{2}=1$$

When the electric‐field direction was designed along the *x*‐axis or *y*‐axis and the light propagated through the crystal whose length was *L* in the *z*‐direction, the phase difference between the component of light in the *x*´direction and that in the *y*´direction was expressed as follows:

(5) $$\Gamma =\frac{2\pi }{\lambda }n_{o}^{3}\gamma_{11}\frac{V}{d}L$$

where n_o_ is ordinary refractive indices at different wavelengths *λ*, *L* is the length of the crystal that was propagated by the light, *d* denotes the thickness of the crystal in the electric‐field direction and *V* represents the external direct voltage.

## Conflict of Interest

The authors declare no conflict of interest.

## Author Contributions

D.Z.L., H.H.Y., and H.J.Z. conceived the project. J.F.H. and Y.Z.W. performed the crystal growth and characterization. J.F.H. performed device processing and laser experiments. F.L. performed the first principles calculations. K.W. performed single‐crystal XRD analysis. All authors co‐wrote and revised the manuscript.

## Supporting information



Supporting Information

## Data Availability

The data that support the findings of this study are available in the supplementary material of this article.
