# Supplementary material for: Electron‐Lattice Synergistic Coordination for Boosting the Electro‐Optic Response of the Crystal
Source: Adv Sci (Weinh). 2024 Dec 5;12(4):2411143. doi: 10.1002/advs.202411143 (PMC11775533; doi:10.1002/advs.202411143)
Supplement: Supplementary file 1 — Supporting Information [file ADVS-12-2411143-s001.docx]

Supporting Information

**Electron-lattice** **synergistic coordination for boosting the electro-optic response of the crystal**

Jinfeng Han^1^, Yuzhou Wang^1,2^, Fei Liang^1^, Kui Wu^1^, Dazhi Lu^1*^, Deliang Cui^1^, Haohai Yu^1**^, Huaijin Zhang^1^

^1^ State Key Laboratory of Crystal Materials and Institute of Crystal Materials, Shandong University, Jinan 250100, China

^2^ The 46^th^ Research Institute, China Electronics Technology Group Corporation, Tianjin 300220, P. R. China

**Corresponding authors:**

* Email address: dazhi.lu@sdu.edu.cn, ** haohaiyu@sdu.edu.cn

**Keywords:** Electro-optic response, Electron-lattice synergistic coordination strategy, Langasite family crystal, Broadband electro-optic modulation

**Table of Contents**

1. **Experimental methods**
2. **Measurement methods**
3. **Calculation Methods**
4. **Figure S1**. PXRD patterns of the LZGS
5. **Figure S2.** (a) The IRRS and *F(ω)*, (b) Raman spectrum and *dS/dω* of LGS powder.
6. **Figure S3.** Crystal crystallinity for different excess ratio of Ga_2_O_3_ compound.
7. **Figure S4.** The state of the remaining material after crystal growth of nitrogen with different oxygen content.
8. **Figure S5.** (a)-(c) Photographs of grown crystals at different thickness temperature fields. The temperature field with different thicknesses: (d)–(f) the temperature gradient of the furnace (ΔT = 80 K); (g)–(i) the von Mises stress distribution of the LZGS crystal. The red curve in (g)–(i) represents the von Mises stress of 3 × 10^6^ Pa in thermal distribution.
9. **Figure S6.** Photographs of (a) LZGS crystal and (b) E-O component.
10. **Figure S7.** (a) High-resolution X-ray diffraction pattern of the (0001) wafer of LZGS crystal; (b) High-resolution X-ray diffraction pattern of the wafer of LZGS crystal.
11. **Figure S8.** The conoscopic interference patterns of the LZGS crystal: (a) the top part, (b) the middle part, and (c) the bottom part.
12. **Figure S9.** Sketches for (a) KDP and (b) LZGS samples used in NLO susceptibility component measurements.
13. **Figure S10.** (a) Experimental data (black line), calculated data (red line), and envelope (blue line) of the Maker fringe pattern based on the *d*_11_ of LZGS crystal. (b) Experimental data (black line), calculated data (red line), and envelope (blue line) of the Maker fringe pattern based on the *d*_36_ of KDP crystal.
14. **Figure S11.** Experimental configuration for E-O coefficient measurement at 633 nm laser.
15. **Figure S12.** Experimental configuration for E-O coefficient measurement at 1991 nm laser.
16. **Figure S13.** Pockels cell of LZGS E-O Q-switch.
17. **Figure S14.** Schematic of the LD pumped LZGS E-O Q-switched laser.
18. **Figure S15.** Schematic of the LD pumped LZGS E-O Q-switched Pr:YLF laser.
19. **Figure S16.** Schematic of the LD pumped LZGS E-O Q-switched Tm:YAP laser.
20. **Figure S17.** (a) and (b) are the different directions and average resistivity in different Fermi levels of LGS and LZGS crystals calculated, respectively. (c) The value of resistivity in the range of Fermi level -0.05~0.05 eV. (The ordinate contains its relaxation time.)
21. **Figure S18.** The refractive index ellipsoidal change of LZGS crystal perpendicular to the Z-axis section after applying a voltage in Y direction.
22. **Figure S19.** The polarization state of linearly polarized light passing through LZGS crystal.
23. **Table S1.** The calculated “flexibility index” *F* of LZGS and LGS.
24. **Table S2.** The curve fitting results *M*(*γ*) of IRRS of LZGS powder.
25. **Table S3.** The curve fitting results *P*(*γ*) of Raman spectrum of LZGS powder.
26. **Table S4.** Contributions of lattice vibration in calculated E-O coefficient of LZGS.
27. **Table S5.** The curve fitting results *M*(*γ*) of IRRS of LGS powder.
28. **Table S6.** The curve fitting results *P*(*γ*) of Raman spectrum of LGS powder.
29. **Table S7.** Contributions of lattice vibration in calculated E-O coefficient of LGS.
30. **Table S8.** Refractive indices of LGS and LZGS crystal.
31. **Table S9.** Dielectric constant and piezoelectric strain constant test results.
32. **Table S10.** Electro-optic performance comparison of LZGS and other proven E-O crystals.
33. **Table S11.** Crystal data and structure refinements of La_3_Zr_0.5_Ga_5_Si_0.5_O_14._
34. **Table S12.** Fractional atomic coordinates (×10^4^) and equivalent isotropic displacement.
35. **Table S13.** Anisotropic displacement parameters (Å^2^ × 10^3^) for La_3_Zr_0.5_Ga_5_Si_0.5_O_14_.
36. **Table S14.** Selected bond lengths for La_3_Zr_0.5_Ga_5_Si_0.5_O_14_.
37. **Table S15.** Selected bond angles for La_3_Zr_0.5_Ga_5_Si_0.5_O_14._
38. **References**

**Experimental methods**

Single Crystal X-ray Determination

A Bruker SMART APEX II 4K CCD single crystal diffractometer was applied to collect the single-crystal X-ray diffraction measurements for La_3_Zr_0.5_Ga_5_Si_0.5_O_14_ under Mo Kα radiation (*λ* = 0.71073 Å) at 298 K. Data collection, reduction, and cell refinement were applied using the software APEX3. The crystal structures were solved by the direct method by intrinsic phasing with the ShelXT structure solution program and refined using least-squares minimization with the ShelXTL refinement package in Olex2. Potential missing symmetry of crystal data was checked with the program PLATON30, and no higher symmetry was found. The detailed crystallographic data for La_3_Zr_0.5_Ga_5_Si_0.5_O_14_ is listed in Table S11-S15.

High-resolution X-ray diffraction

High-resolution X-ray diffraction (HRXRD) was implemented on a Bruker-AXS D5005HR diffractometer equipped with a two-crystal Ge (220) monochromator set for Cu-Kα1 radiation (λ = 1.54056 Å). The accelerating voltage and tube current were 30 kV and 30 mA, and the step time and step size were 0.1 s and 0.001°, respectively. The rocking curves of the (0001) and $\left(11\bar{2}0\right)$ wafers polished to optical quality with the thickness of 2 mm were obtained.

Conoscopic interference patterns

Conoscopic interference patterns were observed by Axio Lab A1 Zeiss Polarized optical microscopy. The grown crystals were processed into 10 × 10 × 2.2 mm^3^ thin sheets and polished on large surfaces.

Resistivity Measurment

The X-cut crystal samples with a dimension of 5 × 5 × 1 mm^3^ were prepared from LGS and LZGS crystals to evaluate the electrical resistivity *ρ_11_* and *ρ_33_* at room temperature. Frist, apply a constant voltage of 1000 V to both ends of the crystal, and calculate the resistance by measuring the current flowing through the sample using Ohm's law. The resistivity is then calculated based on the dimensions of the sample.

**Measurement methods**

1. E-O coefficient measuring method of point group 32

The crystal belonged to point group 32 in the trigonal system with two independent E-O coefficients: *γ_11_* and *γ_41_*. The three principal axes are in the same direction as the physical axes in the crystal. The crystal without the electric field and the refractive-index ellipsoid can be expressed as follows:

$\frac{1}{n_{o}^{2}}\cdot\left(x^{2}+y^{2}\right)+\frac{1}{n_{e}^{2}}\cdot z^{2}=1$ (1)

With the role of the electric field E = (E_1_, E_2_, E_3_), the refractive-index ellipsoid was:

$\left(\frac{1}{n_{o}^{2}}+\gamma_{11}E_{1}\right)\cdot x^{2}+\left(\frac{1}{n_{o}^{2}}-\gamma_{11}E_{1}\right)\cdot y^{2}+\frac{1}{n_{e}^{2}}\cdot z^{2}-2\gamma_{11}E_{2}\cdot xy+2\gamma_{41}E_{1}\cdot yz-2\gamma_{41}E_{2}\cdot xz=1$ (2)

The electric-field direction was designed along the *x*-axis and the refractive-index ellipsoid would be:

$\left(\frac{1}{n_{o}^{2}}+\gamma_{11}E_{1}\right)\cdot x^{2}+\left(\frac{1}{n_{o}^{2}}-\gamma_{11}E_{1}\right)\cdot y^{2}+\frac{1}{n_{e}^{2}}\cdot z^{2}+2\gamma_{41}E_{1}\cdot yz=1$ (3)

Under the action of the electric field, the optical properties of the crystal changed into those of a biaxial crystal. The direction of the new principal refractive index is almost the same as that of the original refractive index. The new principal refractive indices were expressed as:

$\left\{\begin{matrix} n_{1}=n_{o}-\frac{1}{2}n_{o}^{3}\gamma_{11}E_{\text{x}} \\ \\ n_{2}=n_{o}+\frac{1}{2}n_{o}^{3}\gamma_{11}E_{\text{x}} \\ \\ n_{3}=n_{e} \end{matrix}\right.$ (4)

The electric-field direction was designed along the *y*-axis and the refractive-index ellipsoid would be:

$\frac{1}{n_{o}^{2}}\cdot x^{2}+\frac{1}{n_{o}^{2}}\cdot y^{2}+\frac{1}{n_{e}^{2}}\cdot z^{2}-2\gamma_{11}E_{2}\cdot xy-2\gamma_{41}E_{2}\cdot xz=1$ (5)

The direction of the new principal refractive index is rotated 45° around the *z* axis. The relation of the new coordinate system to the original coordinate system is showed in Figure S18. The new principal refractive indices were expressed as:

$\left\{\begin{matrix} n_{1}=n_{o}-\frac{1}{2}n_{o}^{3}\gamma_{11}E_{\text{y}} \\ \\ n_{2}=n_{o}+\frac{1}{2}n_{o}^{3}\gamma_{11}E_{\text{y}} \\ \\ n_{3}=n_{e} \end{matrix}\right.$ (6)

When the light propagated through the crystal whose length was *L* in the Z direction, the phase difference between the component of light in the *x*´direction and that in the *y*´direction was expressed as follows:

$\Gamma=\frac{2\pi}{\lambda}n_{o}^{3}\gamma_{11}\frac{V}{d}L$ (7)

where *n_o_* is ordinary refractive indices at different wavelengths *λ*, *L* is the length of the crystal that was propagated by the light, *d* denotes the thickness of the crystal in the electric-field direction and *V* represents the external direct voltage. Thus, the E-O coefficient will be obtained if the phase difference can be measured under a certain voltage.

1. E-O coefficient measuring method of langasite family

Method 1: The measuring device diagram was shown in the Figure S11. Since langasite family have optical rotation properties, so the beam of light needs to pass through the E-O crystal twice to eliminate its optical rotation. Therefore, the phase difference changed twice through the E-O crystal was expressed as follows:

$\Gamma=\frac{4\pi}{\lambda}n_{o}^{3}\gamma_{11}\frac{V}{d}L$ (8)

First, the polarizer is turned to make make the light intensity zero. When a quarter wave voltage is added to the crystal, the light intensity through the polarizer is maximum, and when half-wave voltage is added, the light intensity through the polarizer is minimum. A quarter wave voltage of the E-O crystal can be expressed as:

$V_{\lambda/4}=\frac{d}{L}\frac{\lambda}{4\gamma_{11}n^{3}}$ (9)

The E-O coefficient $\gamma_{11}$can be calculated according to formula (9).

Method 2: As shown in Figure S12, the laser output through the filter is linearly polarized light, and its wavelength is 1991 nm. The linearly polarized light P_1_ with 45° from the X/Y axis entered the LZGS E-O crystal along the *c*-axis. When the voltage was not applied, the light intensity measured through the crystal was denoted as *I*_0_. Since langasite family had optical rotation characteristics, it was assumed that the linear polarization direction is rotated *α* degree after passing through the crystal. We can understand that linearly polarized light P_2_ is incident on a non-optically active E-O crystal at *θ* = (45-*α*) degrees from X axis (shown in Figure S19), and then the analyzer was turned to make it extinction.

In the case of applied voltage to X axis, the E-O crystal can be regarded as a wave wafer, and the light intensity through the analyzer can be expressed by the following formula:

$I=I_{0}\sin^{2}(2\theta)\cdot\sin^{2}(\text{δ}_{\text{c}}/2)$ (10)

*I_0_* is the intensity of the incident polarized light when the voltage is not applied, and *θ* is the angle between the polarization direction of linear polarized light P_2_ and the new optical axis, and *δc* is the phase difference generated after passing through the LZGS crystal. Among them:

$\text{δ}_{\text{c}}=\frac{2\pi}{\lambda}(n'_{1}-n'_{2})\cdot L$ (11)

$n'_{1}-n'_{2}=n_{o}^{3}\gamma_{11}E$ (12)

$E=\frac{V}{d}$ (13)

The above λ represented the wavelength of the incident light, *L* was E-O crystal light length, *n_o_* was the ordinary optical refractive index of the crystal at wavelength *λ*, *γ_11_* was the E-O coefficient of the LZGS crystal, E was the electric field strength, *V* was the voltage added at both ends of the E-O crystal, *d* is the crystal thickness in the direction of voltage. Combined with the formula (10) (11)(12)(13), the relationship between light intensity transmittance and applied voltage can be expressed as:

$T=\frac{I}{I_{0}\left(\sin2\theta\right)^{2}}=\sin^{2}\frac{\pi}{\lambda}n_{o}^{3}\gamma_{11}V\frac{L}{d}$ (14)

**Calculation Method**

1. The first principles calculations to calculate the conductivity

We have employed the first-principles tool——Vienna Ab initio Simulation Package(VASP)^[1,2]^ to perform all density functional theory (DFT) calculations within the generalized gradient approximation (GGA) using the Perdew-Burke-Ernzerhof (PBE)^[3]^ formulation. We have chosen the projected augmented wave (PAW) potentials^[4,5]^ to describe the ionic cores and take valence electrons into account using a plane wave basis set with a kinetic energy cutoff of 450 eV. Partial occupancies of the Kohn−Sham orbitals were allowed using the Gaussian smearing method and a width of 0.05 eV. For the optimization of both geometry and lattice size, the Brillouin zone integration was performed with 0.02 Å^-1^ *Γ*-centered *k*-point sampling^[6]^. The self-consistent calculations applied a convergence energy threshold of 10^-7^ eV. The equilibrium geometries and lattice constants were optimized with maximum stress on each atom within 0.0004 eV Å^-1^. The weak interaction was described by DFT+D3 method using empirical correction in Grimme’s scheme^[7,8]^. The transport properties were calculated by the tool vaspkit ^[9]^.

1. The first principles calculations to calculated electronic band structure total and partial DOS curves

First-principle calculations were performed using the pseudopotential^[10]^ method implemented in the CASTEP package^[11]^ based on density functional theory (DFT).^[12]^ The optimized norm-conserving pseudopotential^[13]^ were used to simulate the ion-electron interactions for all constituent elements. A kinetic energy cutoff of 940 eV was chosen with Monkhorst−Pack k-point meshes spanning < 0.04/Å^3^ in the Brillouin zone.^[14]^ The cell parameters and atomic positions in the unit cells of all crystals were fully optimized using the BFGS method.^[14]^ The convergence thresholds between optimization cycles for energy change, maximum force, maximum stress, and maximum displacement were set as 5.0 × 10^−6^ eV/atom, 0.01 eV/Å, 0.02 GPa, and 5.0 × 10^−4^ Å, respectively. The optimization was terminated when all of these criteria were satisfied. Then, it should be emphasized that the generalized gradient approximation (GGA) method with the PBE functional^[15]^ usually heavily underestimates the bandgap energy E_g_, while the hybrid HSE06 method can make accurate predictions for UV oxides.^[16]^ Herein, the scissors-corrected^[17]^ GGA method was employed to calculate the optical properties, where the scissors operator was set as the difference between the HSE06 and GGA bandgaps. This self-consistent ab initio approach has been proven to be an efficient way to investigate linear and NLO properties in many types of NLO materials without introducing any experimental parameters.^[18]^

1. Theoretical calculation of the contribution of the lattice vibration.

The E-O coefficient γ was related to the linear E-O susceptibility *χ*^(2)^(-*ω, ω,*0). Further, the contribution of the lattice vibration of *χ*^(2)^(-*ω, ω,*0) corresponded to the linear susceptibility *χ*^(1)^(*ω*) and transition probability of Raman scattering *W*, which can be obtained from the IRRS and Raman spectrum, respectively. The *M*(*γ*) and *P*(*γ*) as the dipole transition matrix element and the transition-susceptibility matrix element were associated with *χ*^(1)^(*ω*) and *W*, respectively. These two matrix elements can be expressed as follows:^[19-21]^

(15)

(16)

In Equation (15), *f*(*ω*) denotes the local field correlation; *ω_i_* and *y_i_* represent each center frequency and the peak width of the relevant fitted peaks, respectively. In Equation (16), *N* denotes the number of particles; *ω_1_* and *ω*_2_ represent the incident light frequency and scattered light frequency of the Raman scattering.

From the IRRS, the relationship between reflectance *R*(*k*) and wave numbers *k* can be acquired. The complex amplitude reflectance $\hat{\gamma}$ and phase shifts *φ*(*k*) are expressed in terms of the Kramers–Kronig relation as:

(17)

(18)

Thus, the relationship between the complex amplitude reflectance $\hat{\gamma}$ and reflectance *R*(*k*) can be derived. the dielectric function and the refractive index, defined $\hat{\varepsilon}\left(\omega\right)\text{=}\varepsilon'\left(\omega\right)\text{+i}\varepsilon''\left(\omega\right)$ and $\hat{n}\left(\omega\right)=n\left(\omega\right)-i\kappa\left(\omega\right)$, respectively, can be described with reflectance *R*(*k*) based on Fresnel’s formula $\hat{\gamma}=\left(\hat{n}-1\right)/\left(\hat{n}+1\right)$ and the relation $\hat{\varepsilon}=\hat{n}^{2}$. Besides, the relationship between the local field correlation and the dielectric function are represented as $f\left(\omega\right)=\left[\varepsilon'\left(\omega\right)+2\right]/3$ according to the Lorentz model. Consequently, in terms of the linear relation $\hat{\varepsilon}=1+4\pi\chi^{\left(1\right)}$,the linear susceptibility *χ*^(1)^(*ω*) in Equation (15) can be derived from the dielectric function, connecting with reflectance *R*(*k*) from the IRRS. Comparing the derived formula to Equation (15), we can define a function $F\left(\omega\right)$ as:

(19)

The expression of *F*(*ω*) shows agreement with the Lorentzian curves. The peak height *H_i_* can be derived from Equation (19) as follows:

(20)

The Lorentzian curve fitting was introduced in the function *F*(*ω*) to obtain the magnitude of each peak height *H_i_* and peak width *y_i_* of the Lorentzian curves. And the magnitude of *M*(*γ*) can be figured out according to Equation (20), contributing to the calculation of E-O coefficients.

On the analysis of the Raman spectrum, the scattering efficiency of Raman scattering *S* was defined as:^[21]^

$S=\frac{4W}{\left(1+n\right)^{2}N_{i}c}$ (21)

where n denotes the refractive index of the powder sample. The transition probability of Raman scattering *W* is associated with the transition-susceptibility matrix element, *P*(*γ*) according to Equation (16). The particle number *N_i_* of the incident light was fitted to the Bose–Einstein distribution, whose expression is .Combining Equations (21) and (16), we summarized the relationship between *S* and *P*(*γ*) as:

(22)

where *ω* represents the frequency of the incident light under laser excitation. Inspecting Equation (22) carefully, we found that it was also in accordance with the expression of Lorentzian curves. As such, Lorentzian curve fitting was employed in Equation (22) as follow:

$\frac{dS}{d\omega}=\sum_{i} H_{i}\frac{y_{i}^{2}}{\left(\omega-\omega_{i}\right)^{2}+y_{i}^{2}}$ (23)

(24)

Where *ω_i_*, *y_i_* and *H_i_* denote the magnitude of the center frequency, peak width and peak height of each fitted peak, respectively. Since *dS/dω* was derived directly from the relative intensity of the Raman scattering, the values of *ω_i_*, *y_i_* and *H_i_* can be obtained expediently from the Raman spectrum through the fitting process. Accordingly, the magnitude of *P*(*γ*) was calculated according to Equation (24) using the fitted peak parameters. Combining the measurements and analyses of IRRS and Raman spectrum, the calculated E-O coefficient of the contribution of the lattice vibration was given by:

(25)

Where *M*(*γ*) and *P*(*γ*) were derived from Equations (20) and (24), respectively. Here, *ω*_γ_ denotes the corresponding center frequency where the fitted peaks overlapped from functions *F*(*ω*) and *dS/dω*. Only the peaks located at the same center frequency to a certain extent contributed to E-O coefficient *γ*.

**Calculated methods of the flexible factor**^[22]^

It is well known that the electrical polarization caused by an applied electric field is associated with a weak distortion of the ion lattice, which also means that the dipole moment generated is related to the flexibility of the ions in the chemical bond under the external electric field. More flexible chemical bonds can provide a larger E-O effect in the crystal.

We employ a simple flexible dipole model. As shown in Figure 1 in the main text, a chemical bond between two atoms is formed by the overlapping valence electrons (simplified to be point charges). If a valence electron is located in the region between the two atom cores with charges *C_a_* and *C_b_*, its equilibrium distance to *C_a_* (or *C_b_*) is $\sqrt{C_{a}}R/\sqrt{C_{a}}+\sqrt{C_{b}}(\sqrt{C_{b}}R/\sqrt{C_{a}}+\sqrt{C_{b}})$, where *R* is the bond length between the two cores. Thus, the Coulombic interaction of the electron from *C_a_* or *C_b_* is $(\sqrt{C_{a}}+\sqrt{C_{b}})^{2}/R^{2}$. Clearly, the flexibility of the electronic motion within the bond (or the compliance with the dipole moment) is proportional to the charge of the bonding valence electrons and is inversely proportional to the Coulombic interaction with the involved two atomic cores. Accordingly, an empirical “flexibility index” *F* associated with the bonding electrons can be defined as the follows:$F=\frac{\exp[(R_{0}-R_{a})/B]}{(\sqrt{C_{a}}+\sqrt{C_{b}})^{2}/R_{a}^{2}}$, where the numerator is the bond valence charge (from the bond valence sum model), and the denominator is the force binding the bound electrons to the cores. *R_a_* is the average bond length of the group, which contributes mostly to the E-O effect, *R*_0_ is the tabulated ideal bond length when the atom contributes exactly one valence unit to the central atom in the group, and B is an empirical constant, typically 0.37°Å. The *C_a_* (and *C_b_*) values for Ga, Zr, O, are 3, 4, and 6, respectively.

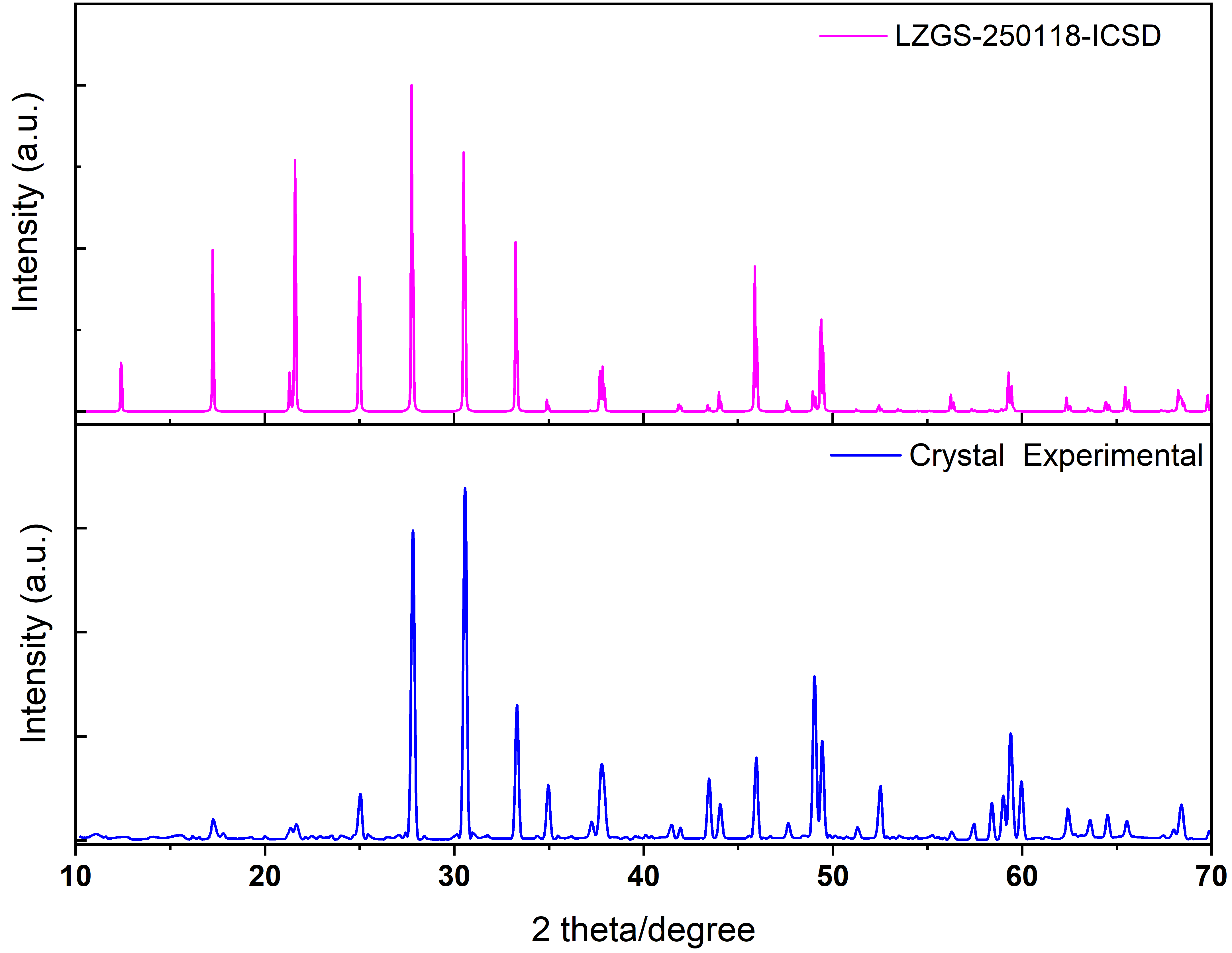


**Figure S1.** PXRD patterns of the LZGS.


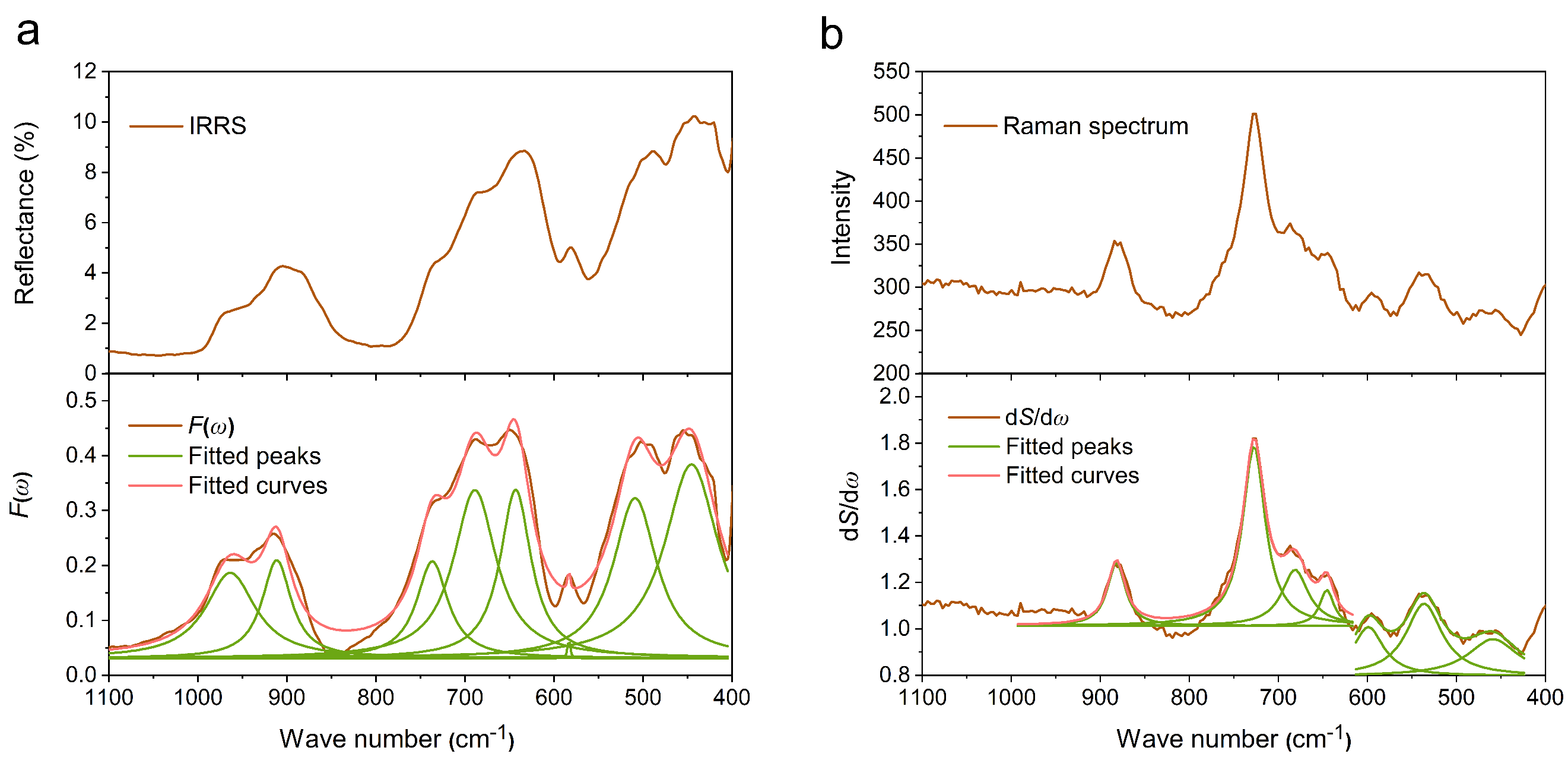


**Figure S2.** (a) The IRRS and *F*(*ω*), (b) Raman spectrum and *dS/dω* of LGS powder.


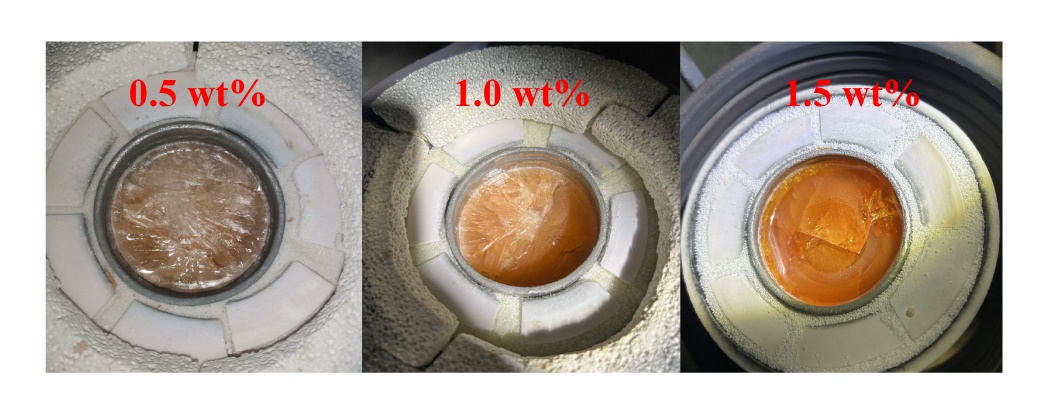


**Figure S3.** Crystal crystallinity for different excess ratio of Ga_2_O_3_ compound.


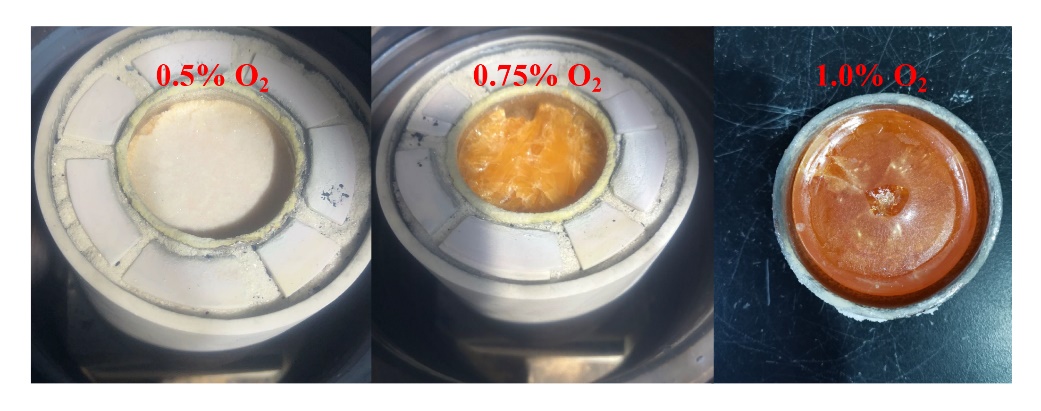


**Figure S4.** The state of the remaining material after crystal growth of nitrogen with different oxygen content.


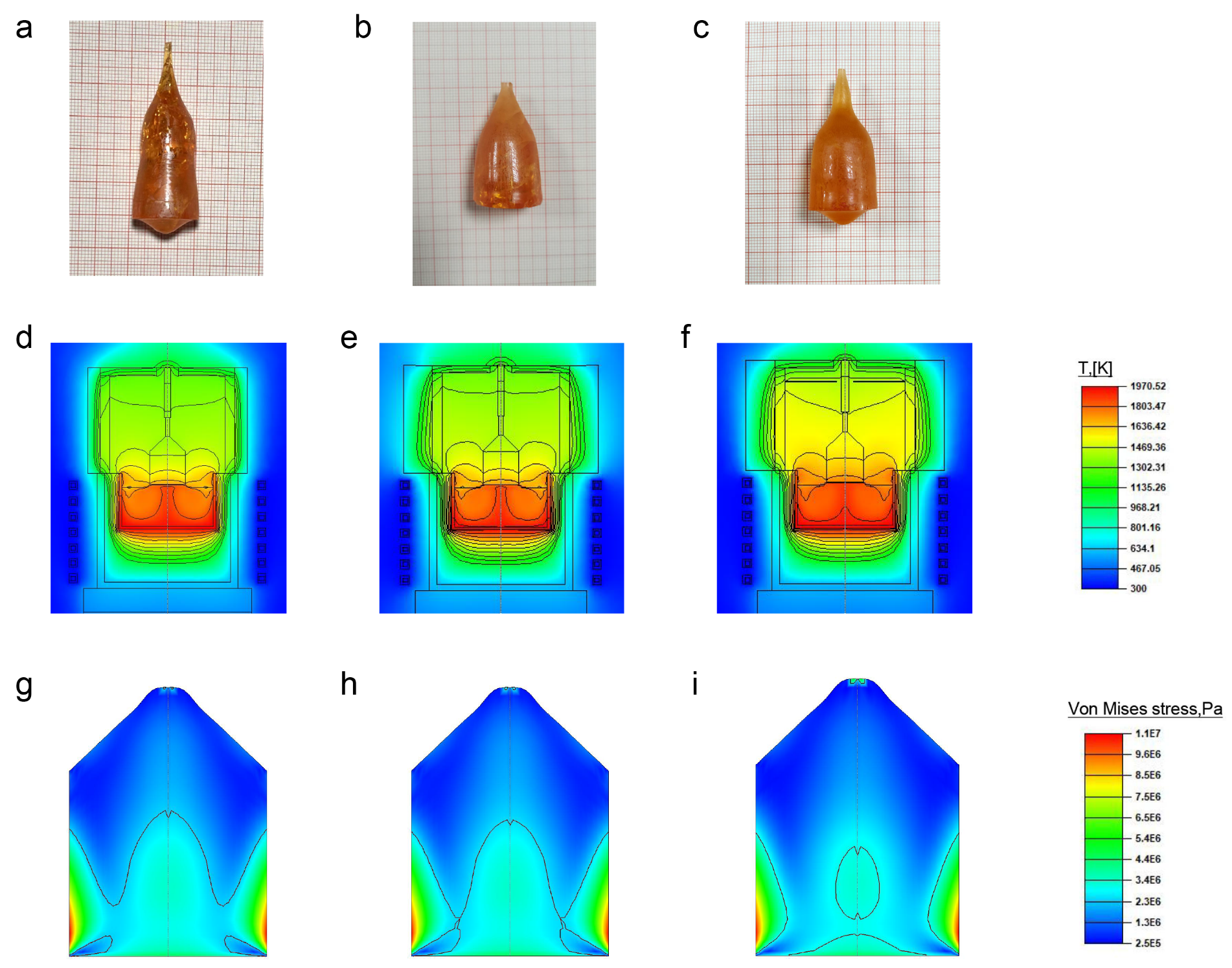


**Figure S5.** (a)-(c) Photographs of grown crystals at different thickness temperature fields. The temperature field with different thicknesses: (d)–(f) the temperature gradient of the furnace (ΔT = 100 K); (g)–(i) the von Mises stress distribution of the LZGS crystal. The red curve in (g)–(i) represents the von Mises stress of 3 × 10^6^ Pa in thermal distribution.

**Growth of LZGS crystal**

The crystals were grown using the Czochralski method, and the [001] direction was selected as the seed crystal, which can effectively avoid spiral growth and have the fastest growth rate. Compared to LGS crystal, the introduction of Zr ion will have an additional influence on the growth of LZGS crystal. First, Compared with LGS crystals, LZGS has more serious volatilization of Ga_2_O_3_ during growth. Then, Zr and Ga ions occupy the B site concurrently, increasing the crystal disorder. In order to solve these crystal growth problems, we optimized the ratio of raw materials, with the ratio of excess Ga_2_O_3_ of 0.5 wt. %, 1 wt. % and 1.5 wt. %, respectively. As shown in Figure S3, the crystal crystallinity of LZGS was improved when Ga_2_O_3_ excess was set as 1.5 wt. %. In addition, we grew LZGS crystal in a nitrogen atmosphere with different oxygen content (0.5%, 0.75%, 1.0%), respectively. When the oxygen content was 1%, the crystallinity of the remaining material was improved significantly (Figure S4). Therefore, Ga_2_O_3_ with an excess ratio of 1.5 wt % and 1% oxygen content was selected in crystal growth to reduce the component deviation.

The temperature field in the furnace is an essential factor in crystal nucleation, growth rate and crystal integrity. The Crystal Growth Simulator (CGSim) package was used for numerical simulation to optimize the temperature gradient in the furnace. Three simulation results demonstrate the relationship between crystal mass and thermal field, as shown in Figure S5. For thin furnace materials, the large thermal gradient in the furnace led to high thermal stress and crystal cracking, especially in the shoulder part of the crystal. Then, the temperature field was thickened, and the cracking in the shoulder was improved compared with that in the thin temperature field. Subsequently, we added an after-heater to the temperature field to reduce the temperature gradient, and the crystal did not crack. Therefore, we chose a thickened temperature field and added an after-heater to minimize the thermal stress and avoid crystal cracks.


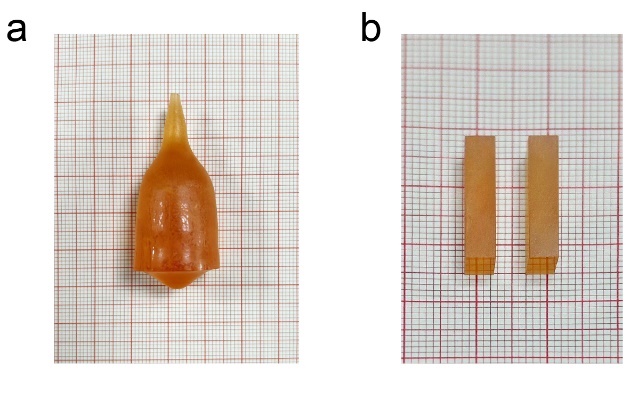


**Figure S6.** Photographs of (a) LZGS crystal and (b) E-O component.


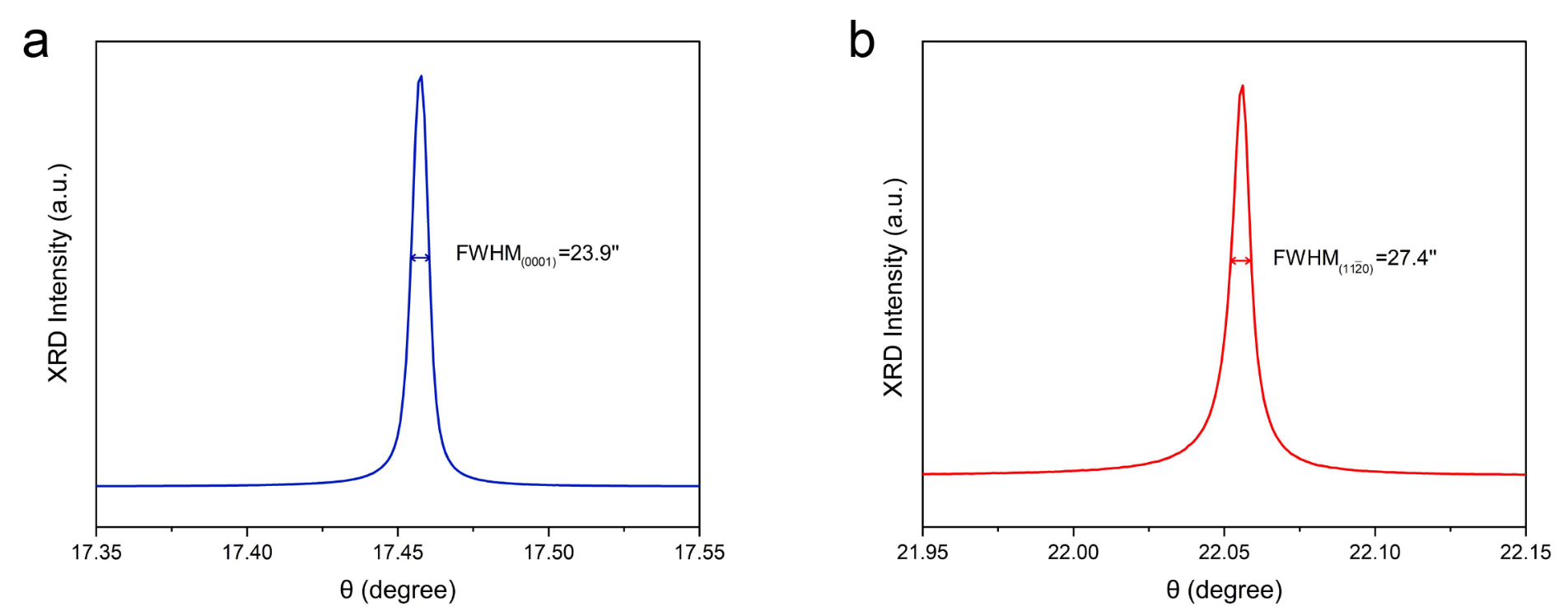


**Figure S7.** (a) High-resolution X-ray diffraction pattern of the (0001) wafer of LZGS crystal; (b) High-resolution X-ray diffraction pattern of the $\left(11\bar{2}0\right)$ wafer of LZGS crystal.

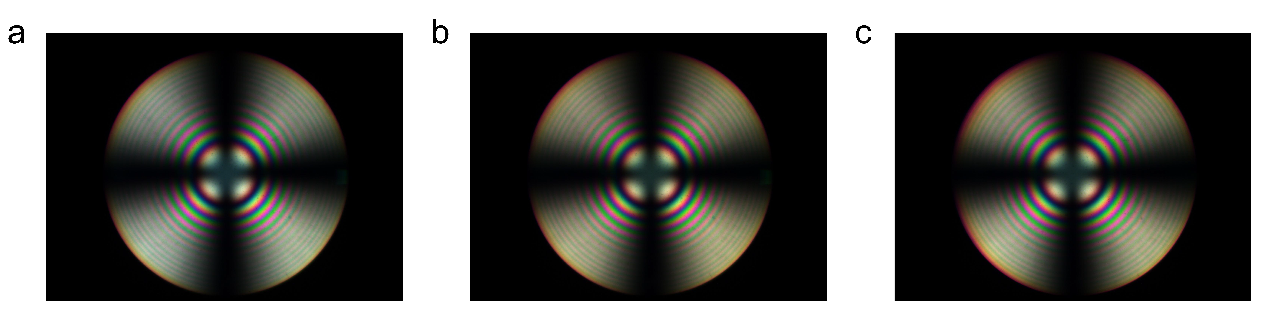


**Figure S8.** The conoscopic interference patterns of the LZGS crystal: (a) the top part, (b) the middle part, and (c) the bottom part.


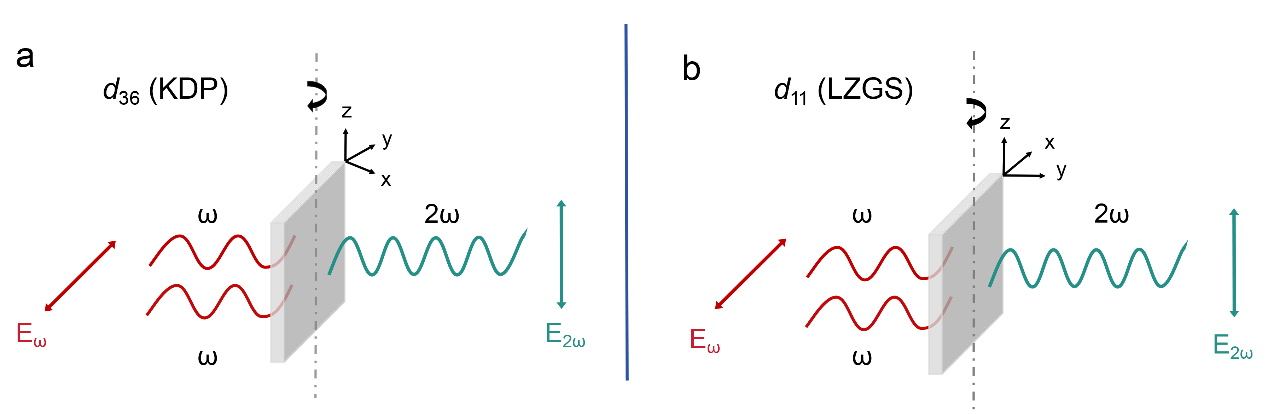


**Figure S9.** Sketches for (a) KDP and (b) LZGS samples used in NLO susceptibility component measurements.


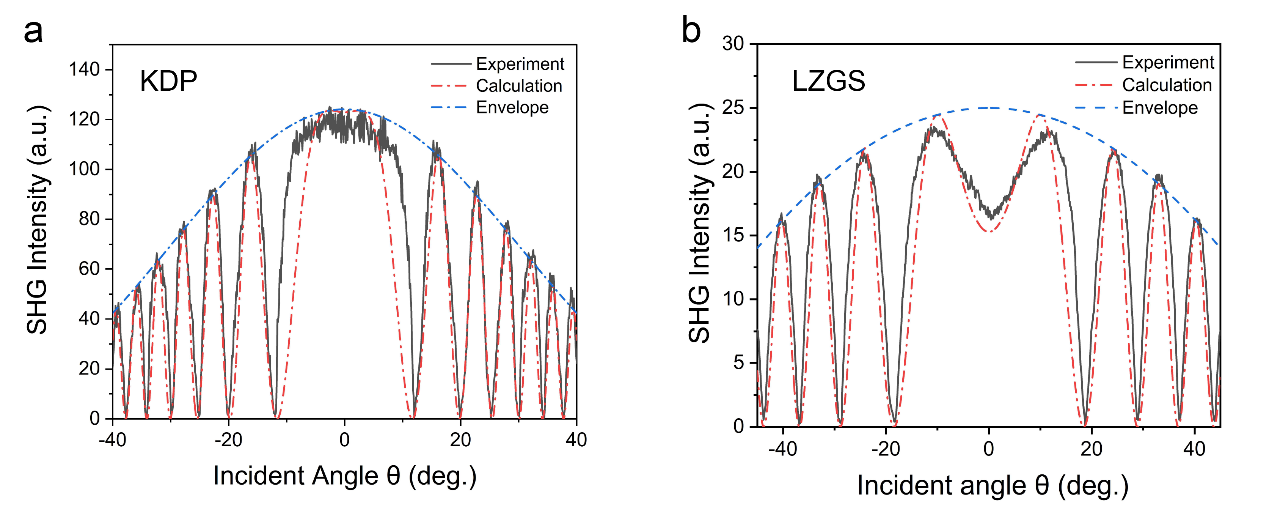


**Figure S10.** (a) Experimental data (black line), calculated data (red line), and envelope (blue line) of the Maker fringe pattern based on the *d*_11_ of LZGS crystal. (b) Experimental data (black line), calculated data (red line), and envelope (blue line) of the Maker fringe pattern based on the *d*_36_ of KDP crystal.


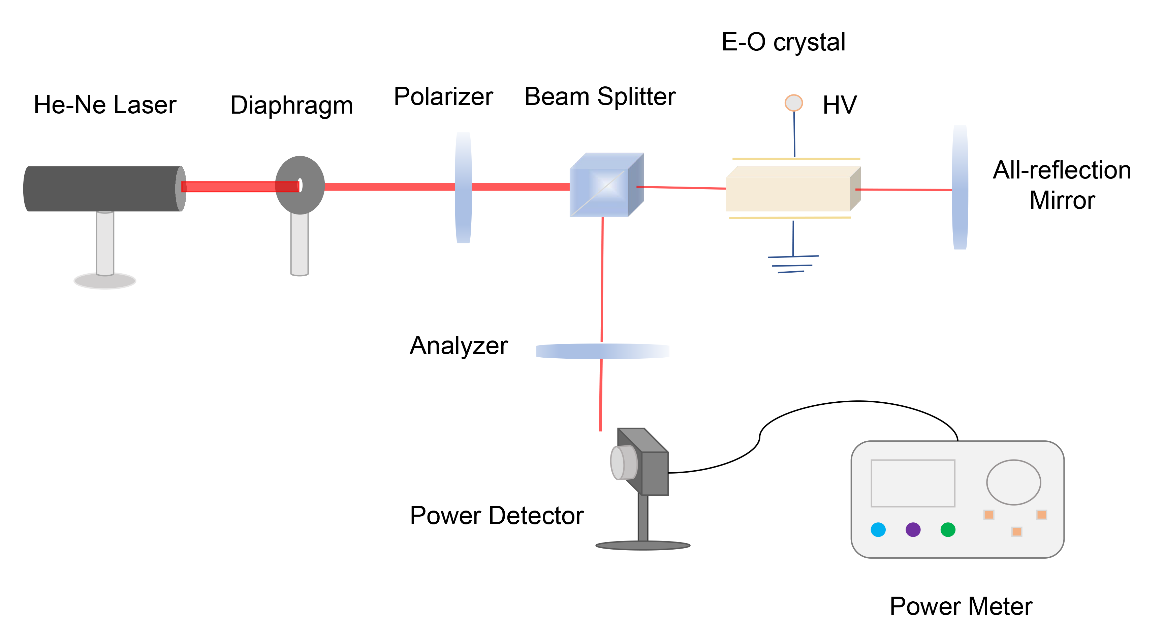


**Figure S11.** Experimental configuration for E-O coefficient measurement at 633 nm laser.


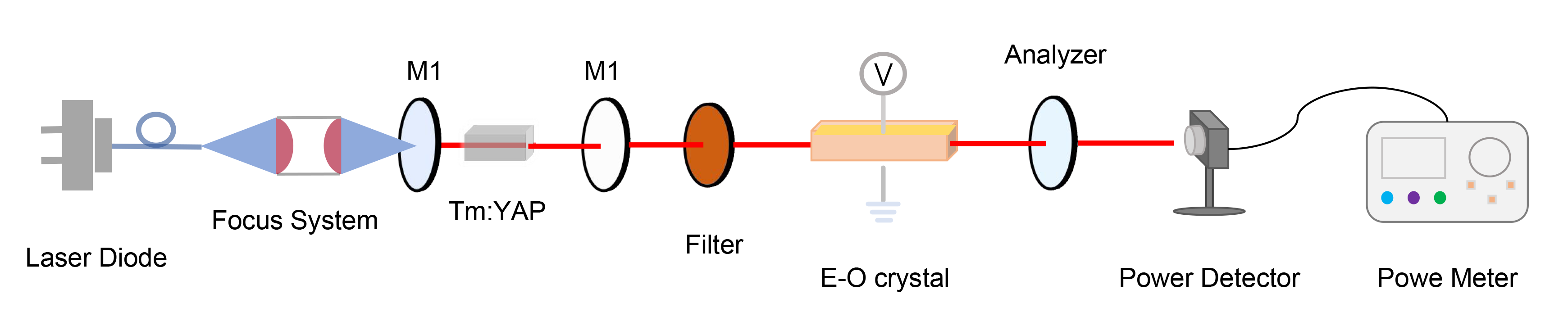


**Figure S12.** Experimental configuration for E-O coefficient measurement at 1991 nm laser.


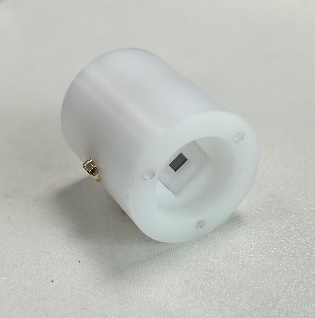


**Figure S13.** Pockels cell of LZGS E-O Q-switch.

**E-O Q-switching experiment at 639 nm**


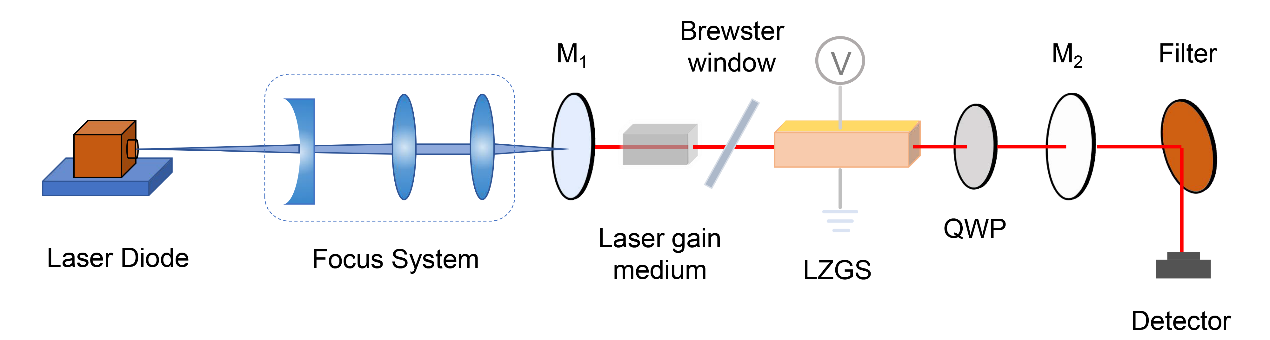


**Figure S14.** Schematic of the LD pumped LZGS electro-optic Q-switched laser.

The pump source was a fiber coupled LD at 444 nm. The laser gain medium was Pr:YLF with a doping concentration of 0.43 at. % and the dimensions for the Pr:YLF was 3 × 3 × 8 mm^3^. The dimension for the Z-cut LZGS E-O crystal was 3 × 3 × 15 mm^3^. The coupling system is the result of our beam shaping, as shown in the Figure S9 for its specific device. The input mirror M_1_ was plane with antireflective (AR) coating at 400-450 nm, and high reflective (HR) coating at 500 - 700 nm. The output mirror M_2_ was concave with a curvature of 200 mm and the transmission (T_oc_) at 640 nm was 1%.

The pump light was focused on the laser crystal Pr:YLF, and the polarizer with uncoated was inserted into the resonator at Brewster angle of 56°, then LZGS E-O crystal that the Y-Z surfaces were coated with Au, and a quarter-wave plate (QWP) were inserted into the laser cavity in order. The quarter wave voltage was 1800 V, according to the theoretical calculation. The laser propagated in the LZGS crystal along the Z axis with the light polarization direction at 45° to the X axis. Because of the optical activity, the quarter wave plate should be rotated to make light disappear, and the Q-switched laser could be achieved by applying a driven voltage with different repetition rates.

**E-O Q-switching experiment at 1064 nm**


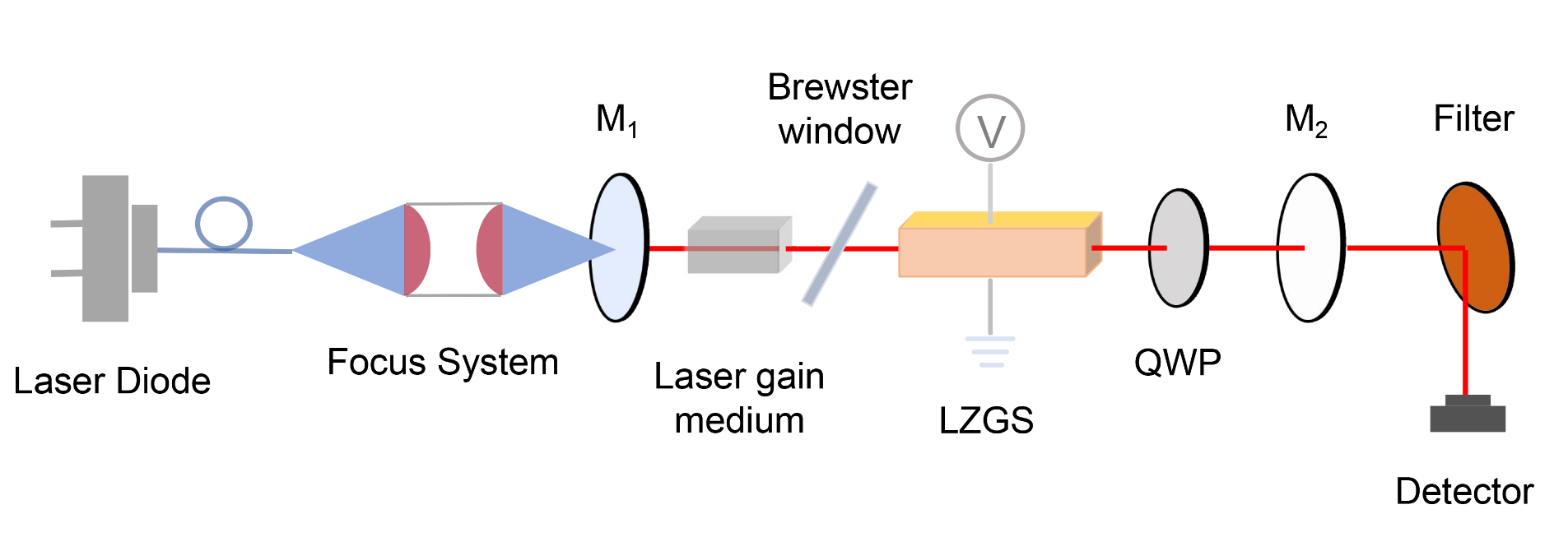


**Figure S15.** Schematic of the LD pumped LZGS electro-optic Q-switched Nd: YVO_4_ laser.

The pump source was a fiber coupled LD at 808 nm. The focusing coupling system with a compression ratio of 1:1, and fiber radius was 100 μm with a numerical aperture of 0.22. The laser gain medium was Nd:YVO_4_ with a doping concentration of 0.5 at. % and the dimensions for the Nd:YVO_4_ is 3 × 3 × 6 mm^3^. The dimension for the LZGS E-O crystal was 3 × 3 × 15 mm^3^ along the Z axis. The input mirror M_1_ was plane with AR coating at 808 nm, and HR coating at 1064 nm. The output mirror M_2_ was concave with a curvature of 200 mm and the T_oc_ at 1064 nm was 15%.

The pump light was focused on the laser crystal Nd:YVO_4_, and the polarizer was inserted into the resonator at Brewster angle of 56°, then LZGS E-O crystal that the Y-Z surfaces were coated with Au, and a QWP were inserted into the laser cavity in order.

**E-O Q-switching experiment at 1991 nm**


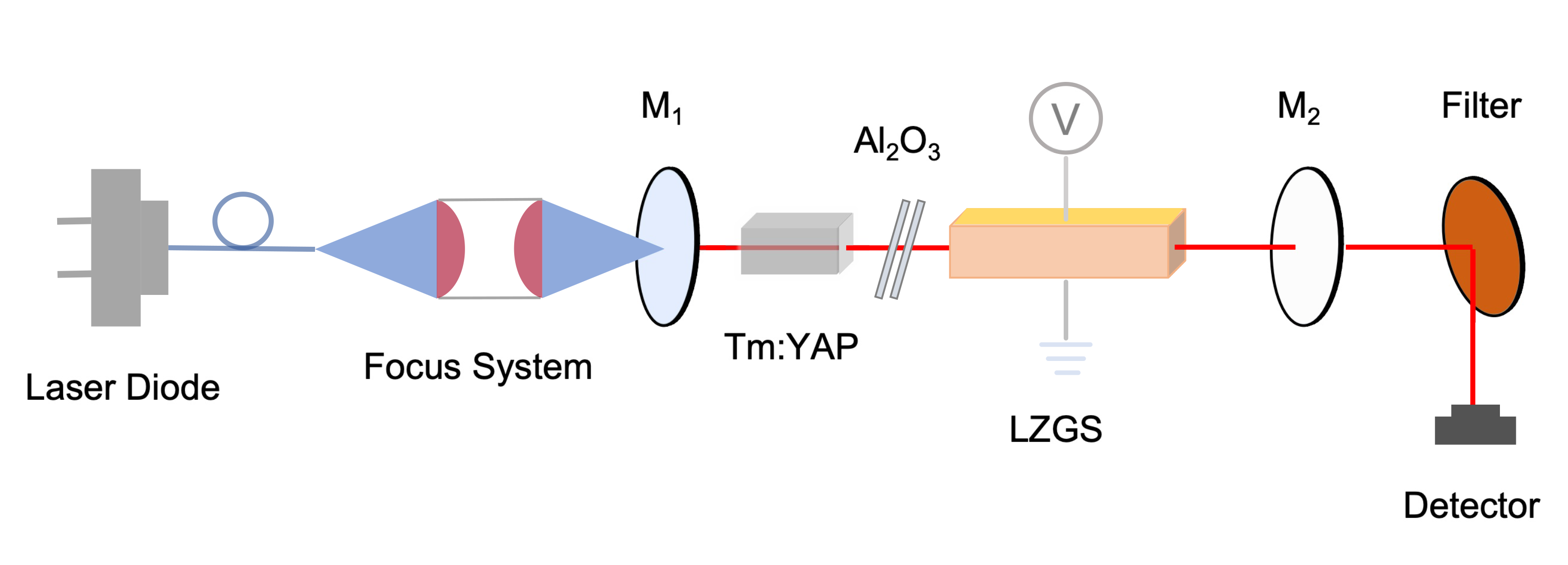


**Figure S16.** Schematic of the LD pumped LZGS electro-optic Q-switched Tm:YAP laser.

The pump source was a fiber coupled LD at 795 nm. The focusing coupling system with a compression ratio of 1:1, and fiber radius was 200 μm with a numerical aperture of 0.22. The gain medium was a 4 at. % doped Tm:YAP crystal with dimensions of 3 × 3 × 10 mm^3^, along *a*-direction. The Z-cut LZGS crystal was in dimensions of 4 × 4 × 20 mm^3^ (X×Y×Z). The coating of M_1_ was AR at 780 – 810 nm and HR at 1850-2100 nm. The output mirror M_2_ was concave and curvature R=200mm. In addition, the T_oc_ at 1850-2100 nm was 20%.

The pump light was focused on the laser crystal Tm:YAP. The polarizer composed of two Al_2_O_3_ plates was inserted into the cavity at the Brewster angle of 56°, and then the LZGS E-O crystal was inserted into the cavity.


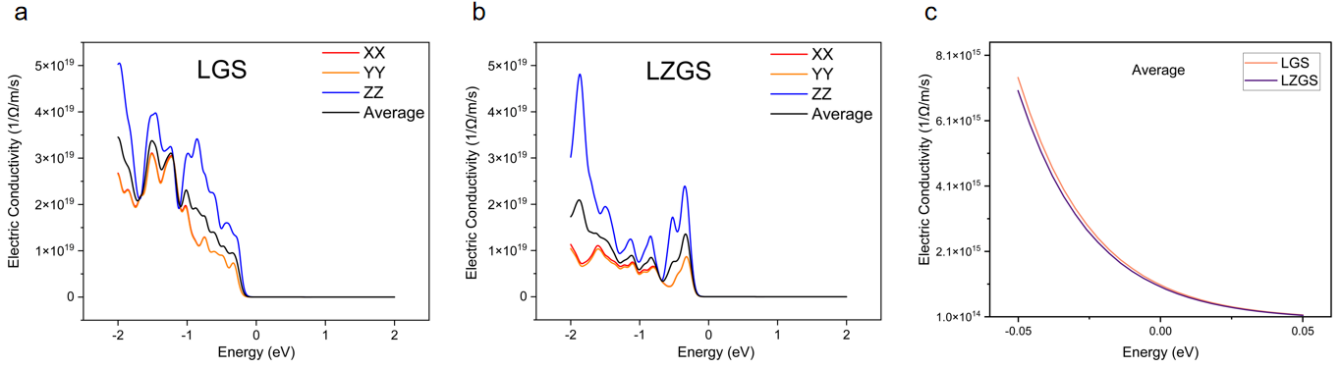


**Figure S17.** (a) and (b) are the different directions and average resistivity in different Fermi levels of LGS and LZGS crystals calculated, respectively. (c) The value of resistivity in the range of Fermi level -0.05~0.05 eV. (The ordinate contains its relaxation time.)

We measured the room temperature resistivity of LZGS and LGS under the same conditions. Test results show that the resistivity of LZGS is *ρ*_11_ = 1.08×10^12^ Ω·cm and that of LGS is *ρ*_11_ = 1.47×10^12^ Ω·cm, and resistivity of the two is comparable. To further verify the accuracy of the measurement, we have employed the first-principles tool——VASP to calculate resistivity of LZGS relative to LGS (Figure S17), and The relative values of the calculated results agree well with the measured results.


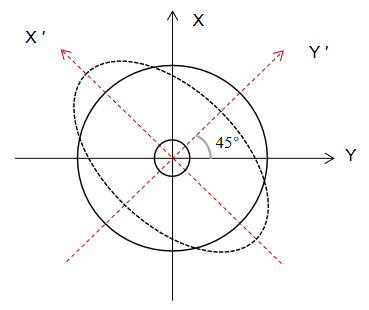


**Figure S18.** The refractive index ellipsoidal change of LZGS crystal perpendicular to the Z-axis section after applying a voltage in Y direction.


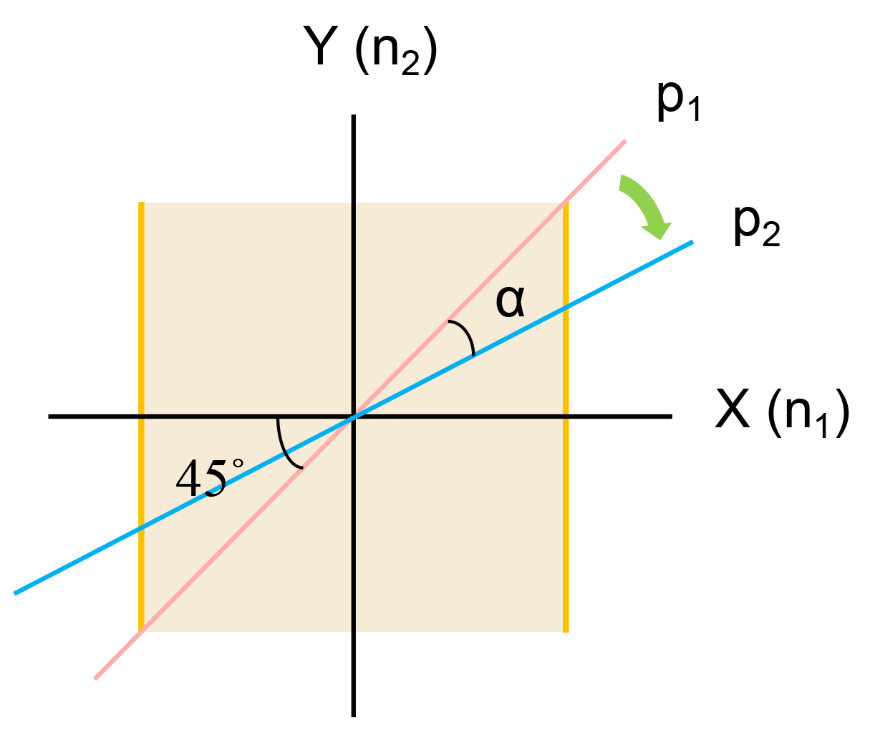


**Figure S19.** The polarization state of linearly polarized light passing through LZGS crystal.

**Table S1.** The calculated “flexibility index” *F* of LZGS and LGS.

|  | M-O (R_0_) | M-O (R_a_) | $\exp[(R_{0}-R_{a})/B]$ | C_a_ (M) | C_b_ (O) | $(\sqrt{C_{a}}+\sqrt{C_{b}})^{2}/R_{a}^{2}$ | *F* |
| --- | --- | --- | --- | --- | --- | --- | --- |
| LZGS | 1.937  (Zr-O) | 2.039 | 0.7591 | 4 | 6 | 4.7620 | 0.1594 |
|  | 1.730  (Ga-O) | 2.039 | 0.4338 | 3 | 6 | 4.2057 | 0.1031 |
|  |  |  |  |  |  |  | 0.1313 |
| LGS | 1.730  (Ga-O) | 1.994 | 0.4899 | 3 | 6 | 4.3976 | 0.1114 |

*R_a_*: average bond length of the group; *R_0_*: ideal bond length; *B*: empirical constant; *C_a_*: cation valence state; *C_b_*: the number of O in a group.

**Table S2.** The curve fitting results *M*(*γ*) of IRRS of LZGS powder.

| Central wavelength  *ω_i_* (cm^-1^) | Peak width  *y_i_* (cm^-1^) | Peak height  *H_i_* | *M*(*γ*)  |
| --- | --- | --- | --- |
| 429.98129 | 36.90521 | 1.35921 | 4.35445×10^-12^ |
| 462.23376 | 20.65587 | 0.61817 | 2.19695×10^-12^ |
| 492.11324 | 56.9516 | 2.56952 | 7.43747×10^-12^ |
| 596.66148 | 30.11738 | 0.92011 | 3.23649×10^-12^ |
| 637.23492 | 76.38663 | 1.35308 | 6.25053×10^-12^ |
| 707.49166 | 61.72354 | 0.20622 | 2.19349×10^-12^ |
| 903.60145 | 30.90074 | 0.40462 | 2.17398×10^-12^ |
| 961.45458 | 49.79124 | 0.30487 | 2.39542×10^-12^ |

**Table S3.** The curve fitting results *P*(*γ*) of Raman spectrum of LZGS powder.

| Central wavelength  *ω_i_* (cm^-1^) | Peak width  *y_i_* (cm^-1^) | Peak height  *H_i_* | *P*(*γ*) |
| --- | --- | --- | --- |
| 432.88754 | 16.75689 | 0.39786 | 7.07319×10^-44^ |
| 511.12121 | 9.74048 | 0.11672 | 4.38611×10^-44^ |
| 580.74237 | 12.14193 | 0.54942 | 9.92755×10^-44^ |
| 665.60947 | 24.82924 | 0.60727 | 2.48395×10^-43^ |
| 701.09229 | 18.76974 | 0.5443 | 2.42556×10^-43^ |
| 867.25046 | 17.17416 | 0.08541 | 7.27123×10^-44^ |

**Table S4.** Contributions of lattice vibration in calculated E-O coefficient of LZGS.

| *M*(*γ*) | *P*(*γ*) |  |
| --- | --- | --- |
| 4.35445×10^-12^ | 7.07319×10^-44^ | 432.88754 |
| 3.23649×10^-12^ | 9.92755×10^-44^ | 580.74237 |
| 6.25053×10^-12^ | 2.48395×10^-43^ | 665.60947 |
| 2.19349×10^-12^ | 2.42556×10^-43^ | 701.09229 |
| 7.43747×10^-12^ | 4.38611×10^-44^ | 511.12121 |

**Table S5.** The curve fitting results *M*(γ) of IRRS of LGS powder.

| Central wavelength  *ω_i_* (cm^-1^) | Peak width  *y_i_* (cm^-1^) | Peak height  *H_i_* | *M*(*γ*) |
| --- | --- | --- | --- |
| 445.58726 | 73.42497 | 0.35353 | 3.13243×10^-12^ |
| 508.97164 | 59.54876 | 0.29204 | 2.56389×10^-12^ |
| 583.34346 | 2.99802 | 0.03853 | 2.08957×10^-13^ |
| 643.1444 | 42.49471 | 0.3073 | 2.22174×10^-12^ |
| 688.92036 | 58.31264 | 0.30664 | 2.59981×10^-12^ |
| 736.79593 | 43.08654 | 0.17676 | 1.69673×10^-12^ |
| 911.45396 | 39.89036 | 0.17909 | 1.64328×10^-12^ |
| 963.46996 | 68.00047 | 0.15593 | 2.00199×10^-12^ |

**Table S6.** The curve fitting results *P*(γ) of Raman spectrum of LGS powder.

| Central wavelength  *ω_i_* (cm^-1^) | Peak width  *y_i_* (cm^-1^) | Peak height  *H_i_* | *P*(*γ*) |
| --- | --- | --- | --- |
| 536.54097 | 35.9531 | 0.22516 | 1.61407×10^-43^ |
| 597.05981 | 25.35566 | 0.1151 | 9.69536×10^-44^ |
| 646.51693 | 14.57336 | 0.13755 | 7.99624×10^-44^ |
| 681.88393 | 31.86979 | 0.22377 | 1.68076×10^-43^ |
| 727.17544 | 28.22905 | 0.75725 | 3.33722×10^-43^ |
| 881.45199 | 18.47212 | 0.25248 | 1.48429×10^-43^ |

**Table S7.** Contributions of lattice vibration in calculated E-O coefficient of LGS.

| *M*(*γ*) | *P*(*γ*) |  |
| --- | --- | --- |
| 3.13243×10^-12^ | 5.00753×10^-44^ | 461.69861 |
| 2.56389×10^-12^ | 1.61407×10^-43^ | 536.54097 |
| 2.08957×10^-13^ | 9.69536×10^-44^ | 597.05981 |
| 2.22174×10^-12^ | 7.99624×10^-44^ | 646.51693 |
| 2.59981×10^-12^ | 1.68076×10^-43^ | 681.88393 |
| 1.69673×10^-12^ | 3.33722×10^-43^ | 727.17544 |
| 1.64328×10^-12^ | 1.48429×10^-43^ | 881.45199 |

**Table S8.** Refractive indices of LGS and LZGS crystal.

| Wavelength  (μm) | LGS | | LZGS | |
| --- | --- | --- | --- | --- |
|  | *n*_e_ | *n*_o_ | *n*_e_ | *n*_o_ |
| 0.4046 | 1.95662 | 1.9455 | 1.98811 | 1.96596 |
| 0.4358 | 1.94552 | 1.93424 | 1.9759 | 1.95421 |
| 0.48 | 1.93381 | 1.92246 | 1.9629 | 1.94142 |
| 0.546 | 1.92177 | 1.91036 | 1.9496 | 1.92874 |
| 0.5876 | 1.9163 | 1.90491 | 1.94393 | 1.92269 |
| 0.6438 | 1.91067 | 1.89925 | 1.93743 | 1.91666 |
| 0.7065 | 1.90577 | 1.8944 | 1.93213 | 1.9118 |
| 0.8521 | 1.89836 | 1.88692 | 1.9243 | 1.90406 |
| 1.014 | 1.89324 | 1.8818 | 1.9183 | 1.8981 |
| 1.5296 | 1.88396 | 1.87265 | 1.9129 | 1.8931 |
| 2.325 | 1.87348 | 1.86232 | 1.90245 | 1.88299 |

**Table S9.** Dielectric constant and piezoelectric strain constant test results.

|  | ε_11_ | ε_33_ | d_11_ (pC·N^-1^) | d_14_ (pC·N^-1^) |
| --- | --- | --- | --- | --- |
| LGS | 18.9 | 47.0 | 6.3 | -5.4 |
| LZGS | 19.4 | 47.8 | 6.1 | -5.1 |

**Table S10.** Electro-optic performance comparison of LZGS and other proven EO crystals.^[23-26]^

|  | DKDP | LiNbO_3_ | KTP | RTP | β-BBO | LGS | LZGS |
| --- | --- | --- | --- | --- | --- | --- | --- |
| Transparency (μm) | 0.2~2.1 | 0.28~5.5 | 0.35~5.5 | 0.35~4.5 | 0.19~3.3 | 0.25~7.2 | 0.26~7.6 |
| Electrical resistivity (Ω·cm) | 10^11^ | 10^13^ | 10^6^~10^12^ | 10^7^~10^11^ | 10^8^~10^14^ | 10^12^ | 10^12^ |
| n^3^γ (pm/V) | 89 | 329 | 234 | 267 | 10 | 15 | 18.3 |
| Laser damage threshold  (GW·cm^-2^ @1064nm,10ns) | ＞5 | 0.3 | 0.6/1.5 | 0.86 | 5 | 1.3 | 1.43 |
| Piezoelectric ringing  Effect (High RF) | stronger | stronger | slight | weaker | weaker | weaker | weaker |
| Deliquescence or not | yes | no | no | no | slight | no | no |
| Optical homogeneity | excellent | normal | good | normal | excellent | good | good |
| Temperature stability | poor | poor | good | good | good | good | good |

**Table S11.** Crystal data and structure refinements of La_3_Zr_0.5_Ga_5_Si_0.5_O_14_.

|  | La_3_Zr_0.5_Ga_5_Si_0.5_O_14_ |
| --- | --- |
| Formula weight | 1046.47 |
| Crystal system | Trigonal |
| Space group | *P*321 |
| *a* (Å) | 8.22010(10) |
| *b* (Å) | 8.22010(10) |
| *c* (Å) | 5.1328(2) |
| *α* (deg) | 90 |
| *β* (deg) | 90 |
| *γ* (deg) | 120 |
| V/Å^3^ | 300.358(14) |
| Z | 1 |
| *ρ*calc(g·cm^-3^ ) | 5.785 |
| *μ* (mm^-1^ )) | 21.912 |
| *F*(000) | 452 |
| Flack parameter | -0.02(4) |
| Reflections collected | 550 |
| Final *R* indexes [I≥2σ(I)] ^[a]^ | *R*1 =0.0263 w*R*2 =0.0568 |
| Final *R* indexes [all data] ^[a]^ | *R*1 =0.0277 w*R*2 =0.0576 |

[a]$\text{R}_{1}=\sum\left|\left|F_{o}\right|-\left|F_{c}\right|\right|/\sum\left|F_{o}\right|,\omega R_{2}=\left[\sum\omega\left(F_{o}^{2}-F_{c}^{2}\right)_{2}/\sum\omega\left(F_{o}^{2}\right)^{2}\right]^{1/2}$

**Table S12.** Fractional atomic coordinates (×10^4^) and equivalent isotropic displacement

Parameters (Å^2^ × 10^3^) for La_3_Zr_0.5_Ga_5_Si_0.5_O_14_.

| Atom | x | y | z | U (eq) ^[a]^ |
| --- | --- | --- | --- | --- |
| La | 4232.9(9) | 10000 | 0 | 10.3(3) |
| Zr | 0 | 10000 | 0 | 10.2(5) |
| Ga1 | 0 | 10000 | 0 | 10.2(5) |
| Ga2 | 0 | 7607.0(18) | 5000 | 11.1(4) |
| Ga3 | -3333.33 | 3333.33 | 4687.(4) | 7.8(7) |
| Si | -3333.33 | 3333.33 | 4687.(4) | 7.8(7) |
| O1 | -1494.(10) | 5390.(10) | 3114.(15) | 17.1(15) |
| O2 | -3333.33 | 3333.33 | 813(2) | 15.(2) |
| O3 | 1439.(10) | 9185.(10) | 2398.(12) | 17.4(15) |

[a] U_eq_ is defined as 1/3 of the trace of the orthogonalised U_ij_ tensor.

**Table S13.** Anisotropic displacement parameters (Å^2^ × 10^3^) for La_3_Zr_0.5_Ga_5_Si_0.5_O_14_.

| Atom | U11 | U22 | U33 | U23 | U13 | U12 |
| --- | --- | --- | --- | --- | --- | --- |
| La | 13.3(4) | 11.2(4) | 5.6(3) | 0.1(3) | 0.06(13) | 5.6(2) |
| Zr | 13.1(7) | 13.1(7) | 4.3(9） | 0 | 0 | 6.6(4) |
| Ga1 | 13.1(7) | 13.1(7) | 4.3(9） | 0 | 0 | 6.6(4) |
| Ga2 | 12.4(7) | 11.9(6) | 9.2(7) | 2.5(3) | 4.9(5) | 6.2(4) |
| Ga3 | 10.1(8) | 10.1(8) | 3.2(9) | 0 | 0 | 5.1(4) |
| Si | 10.1(8) | 10.1(8) | 3.2(9) | 0 | 0 | 5.1(4) |
| O1 | 19.(4) | 14.(4) | 16.(3) | -6.(3) | 4.(3) | 7.(3) |
| O2 | 17.(4) | 17.(4) | 12.(5) | 0 | 0 | 8.6(18) |
| O3 | 15.(4) | 25.(4) | 11.(3) | 10.(3) | 4.(3) | 9.(3) |

**Table S14.** Selected bond lengths for La_3_Zr_0.5_Ga_5_Si_0.5_O_14_.

| Atom 1 | Atom 2 | Length (Å) |
| --- | --- | --- |
| La01 | O003 | 2.387(7) |
| La01 | O003 | 2.387(7) |
| La01 | O001 | 2.477(7) |
| La01 | O001 | 2.477(7) |
| La01 | O002 | 2.636(4) |
| La01 | O002 | 2.636(4) |
| La01 | O001 | 2.901(7) |
| La01 | O001 | 2.901(7) |
| Zr01 | O003 | 2.038(7) |
| Zr01 | O003 | 2.038(7) |
| Zr01 | O003 | 2.038(7) |
| Zr01 | O003 | 2.038(7) |
| Zr01 | O003 | 2.039(7) |
| Zr01 | O003 | 2.039(7) |
| Ga02 | O003 | 1.825(6) |
| Ga02 | O003 | 1.825(6) |
| Ga02 | O001 | 1.878(7) |
| Ga02 | O001 | 1.878(7) |
| Si01 | O002 | 1.769(12) |
| Si01 | O001 | 1.800(7) |
| Si01 | O001 | 1.800(7) |
| Si01 | O001 | 1.800(7) |

**Table S15.** Selected bond angles for La_3_Zr_0.5_Ga_5_Si_0.5_O_14_.

| Atom 1 | Atom 2 | Atom 3 | Angle (°) | Atom 1 | Atom 2 | Atom 3 | Angle (°) |
| --- | --- | --- | --- | --- | --- | --- | --- |
| O003 | La01 | O003 | 69.5(3) | O003 | Zr01 | O003 | 83.7(4) |
| O003 | La01 | O001 | 138.4(3) | O003 | Zr01 | O003 | 103.6(4) |
| O003 | La01 | O001 | 106.9(2) | O003 | Zr01 | O003 | 87.3(3) |
| O003 | La01 | O001 | 106.9(2) | O003 | Zr01 | O003 | 83.7(4) |
| O003 | La01 | O001 | 138.4(3) | O003 | Zr01 | O003 | 87.3(3) |
| O001 | La01 | O001 | 101.6(3) | O003 | Zr01 | O003 | 87.3(3) |
| O003 | La01 | O002 | 127.1(2) | O003 | Zr01 | O003 | 103.6(4) |
| O003 | La01 | O002 | 77.9(2) | O003 | Zr01 | O003 | 165.5(4) |
| O001 | La01 | O002 | 90.0(2) | O003 | Ga02 | O003 | 134.5(5) |
| O001 | La01 | O002 | 72.4(3) | O003 | Ga02 | O001 | 107.9(3) |
| O003 | La01 | O002 | 77.9(2) | O003 | Ga02 | O001 | 100.9(3) |
| O003 | La01 | O002 | 127.1(2) | O003 | Ga02 | O001 | 100.9(3) |
| O001 | La01 | O002 | 72.4(3) | O003 | Ga02 | O001 | 107.9(3) |
| O001 | La01 | O002 | 90.0(2) | O001 | Ga02 | O001 | 99.9(4) |
| O002 | La01 | O002 | 152.32(6) | O002 | Si01 | O001 | 116.6(2) |
| O003 | La01 | O001 | 68.1(2) | O002 | Si01 | O001 | 116.6(3) |
| O003 | La01 | O001 | 79.8(2) | O001 | Si01 | O001 | 101.4(3) |
| O001 | La01 | O001 | 153.53(11) | O002 | Si01 | O001 | 116.6(2) |
| O001 | La01 | O001 | 61.8(3) | O001 | Si01 | O001 | 101.4(3) |
| O002 | La01 | O001 | 65.9(3) | O001 | Si01 | O001 | 101.4(3) |
| O002 | La01 | O001 | 124.6(3) | Si01 | O001 | Ga02 | 121.0(4) |
| O003 | La01 | O001 | 79.8(2) | Si01 | O001 | La01 | 105.9(3) |
| O003 | La01 | O001 | 68.1(2) | Ga02 | O001 | La01 | 119.7(3) |
| O001 | La01 | O001 | 61.8(3) | Si01 | O001 | La01 | 90.9(3) |
| O001 | La01 | O001 | 153.53(11) | Ga02 | O001 | La01 | 110.3(3) |
| O002 | La01 | O001 | 124.6(3) | La01 | O001 | La01 | 104.2(3) |
| O002 | La01 | O001 | 65.9(3) | Si01 | O002 | La01 | 111.3(2) |
| O001 | La01 | O001 | 140.9(3) | Si01 | O002 | La01 | 111.3(2) |
| O003 | Zr01 | O003 | 103.6(4) | La01 | O002 | La01 | 107.6(3) |
| O003 | Zr01 | O003 | 165.5(4) | Si01 | O002 | La01 | 111.3(2) |
| O003 | Zr01 | O003 | 87.3(3) | La01 | O002 | La01 | 107.6(3) |
| O003 | Zr01 | O003 | 87.3(3) | La01 | O002 | La01 | 107.6(3) |
| O003 | Zr01 | O003 | 165.5(4) | Ga02 | O003 | Zr01 | 113.5(3) |
| O003 | Zr01 | O003 | 83.7(4) | Ga02 | O003 | La01 | 140.1(4) |
| O003 | Zr01 | O003 | 87.3(3) | Zr01 | O003 | La01 | 103.4(3) |

**Reference**

[1] G. Kresse, J. Furthmüller, *Comput. Mater. Sci.* **1996**, *6*, 15.

[2] G. Kresse, J. Furthmüller, *Phys. Rev. B* **1996**, *54*, 11169.

[3] J. P. Perdew, K. Burke, M. Ernzerhof, *Phys. Rev. Lett.* **1996**, *77*, 3865.

[4] G. Kresse, D. Joubert, *Phys. Rev. B* **1999**, *59*, 1758.

[5] P. E. Blöchl, *Phys. Rev. B* **1994**, *50*, 17953.

[6] H. J. Monkhorst, J. D. Pack, *Phys. Rev. B* **1976**, *13*, 5188.

[7] S. Grimme, J. Antony, S. Ehrlich, H. Krieg, *J. Chem. Phys.* **2010**, *132*, 154104

[8] S. Grimme, S. Ehrlich, L. Goerigk, *J. Comput. Chem.* **2011**, *32*, 1456.

[9] V. Wang, N. Xu, J. C. Liu, G. Tang, W. Geng, Comput. Phys. Commun. **2019**, *267*, 108033.

[10] M. C. Payne, M. P. Teter, D. C. Allan, T. Arias, a. J. Joannopoulos, *Rev. Mod. Phys.* **1992**, *64*, 1045.

[11] S. Clark, M. Segall, C. Pickard, P. Hasnip, M. Probert, K. Refson, *Z. Kristall.* **2005**, *220*, 567.

[12] W. Kohn, *Rev. Mod. Phys.* **1999**, *71*, 1253.

[13] A. M. Rappe, K. M. Rabe, E. Kaxiras, J. Joannopoulos, *Phys. Rev. B* **1990**, *41*, 1227.

[14] H. J. Monkhorst, J. D. Pack, *Phys. Rev. B* **1976**, *13*, 5188.

[15] J. P. Perdew, K. Burke, M. Ernzerhof, *Phys. Rev. Lett.* **1996**, *77*, 3865.

[16] F. Liang, L. Kang, X. Zhang, M.-H. Lee, Z. Lin, Y. Wu, *Cryst. Growth Des.* **2017**, *17*, 4015.

[17] C. S. Wang, B. M. Klein, *Phys. Rev. B* **1981**, *24*, 3417.

[18] Z. Lin, X. Jiang, L. Kang, P. Gong, S. Luo, M.-H. Lee, *J. Phys. D-Appl. Phys.* **2014**, *47*, 253001.

[19] S. Sanna, S. Neufeld, M. Rüsing, G. Berth, A. Zrenner, W. G. Schmidt, *Phys. Rev. B* **2015**, *91*, 224302.

[20] S. M. Kostritskii, P. Bourson, M. Aillerie, M. Fontana, D. Kip, *Appl. Phys. B-Lasers Opt.* **2006**, *82*, 423.

[21] F. Xu, G. Zhang, M. Luo, G. Peng, Y. Chen, T. Yan, N. Ye, *Natl. Sci. Rev.* **2021**, *8*, nwaa104.

[22] H. Lan, F. Liang, X. Jiang, C. Zhang, H. Yu, Z. Lin, H. Zhang, J. Wang, Y. Wu, *J. Am. Chem. Soc.* **2018**, *140*, 4684.

[23] P. Becker, *Adv. Mater.* **1998**, *10*, 979.

[24] D. N. Nikogosyan, *Nonlinear optical crystals: a complete survey*, Springer Science & Business Media, New York, NY, USA **2006**.

[25] H. Zhou, X. He, W. Wu, J. Tong, J. Wang, Y. Zuo, Y. Wu, C. Zhang, Z. Hu, *Light-Sci. Appl.* **2023**, *12*, 23.

[26] S. Shi, R. Shi, G. Wang, Y. Xiao, H. Wang, J. Chen, *Journal of Synthetic Crystals* **2023**, *52*, 2151.
